# Extracellular‐Matrix‐Based Materials from Decellularized Tissue: Opportunities, Challenges, and Future Directions in Regenerative Medicine

**DOI:** 10.1002/adhm.202502107

**Published:** 2025-11-17

**Authors:** Madeline Laude, Vasiliki Kolliopoulos, Antonios G. Mikos, Lisa J. White, Elizabeth Cosgriff‐Hernandez

**Affiliations:** ^1^ Department of Biomedical Engineering The University of Texas Austin TX 78712 USA; ^2^ Deparment of Bioengineering Rice University Houston TX 77005 USA; ^3^ School of Pharmacy The University of Nottingham Nottingham NG7 2RD UK

**Keywords:** advanced biomanufacturing, decellularized tissue, extracellular matrix, tissue engineering

## Abstract

Extracellular matrix (ECM) biomaterials have been used as inductive scaffolds to promote tissue regeneration in a variety of clinical applications. ECM biomaterials derived from decellularized tissue (dECM)   can retain essential bioactive components of the native ECM that can guide cellular processes; however, the composition and bioactivity of the dECM is highly dependent on the decellularization and postprocessing methods. This intrinsic regenerative potential underpins the clinical success of dECM biomaterials in applications including soft tissue repair, cardiac reconstruction, and urological interventions. Here, clinical use of current dECM biomaterials, advances in the fabrication of dECM biomaterials, and hurdles to clinical translation of dECM products are discussed. Clinical use to date has mostly been limited to native dECM sheets or milling to generate powder dECM. Recent advances in fabrication methods from electrospinning to 3D printing have expanded the potential clinical applications of dECM biomaterials by increasing the structural and compositional complexity available to researchers. Despite significant progress, challenges remain in standardizing decellularization processes, optimizing dECM bioactivity retention, and ensuring mechanical compatibility with native tissues. Future research should focus on refining fabrication techniques and establishing standardized criteria for dECM characterization and translational pathways.

## Graphical abstract

Extracellular matrix‐based materials derived from decellularized tissue (dECM) harness the tissue's native bioactivity to guide repair and regeneration across diverse clinical applications. This perspective highlights clinical uses of dECM biomaterials and advances in fabrication methods such as electrospinning and 3D printing. It also addresses hurdles to clinical translation, offering insights into the future of dECM in regenerative medicine.

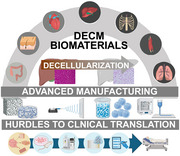

## 1 Introduction

Biomaterial scientists have worked for decades to recapitulate the structure and function of complex tissues in order to fabricate devices that can restore function and guide regeneration. Biomaterials are designed to mimic the native tissue function and mechanical properties while allowing for native cell infiltration, differentiation, and extracellular matrix (ECM) deposition.^[^ ^1^, ^2^ ^]^ However, no synthetic material has approached the regenerative potential of native tissues because of the failure to recapitulate the complex microenvironment and composition of the native ECM.^[^ ^1^, ^3^ ^]^ In order to capture this capacity for repair, researchers have developed methods to decellularize tissues to remove the immunogenic components while retaining the factors that support tissue repair and regeneration.^[^ ^4^, ^5^, ^6^, ^7^ ^]^ These extracellularmatrix‐based biomaterials derived from decellularized tissues (dECM) have shown clinical success in simple form factors that match the native tissue (e.g., MatriStem, DermACELL, OASIS, Alloderm) or powders (e.g., MicroMatrix, OASISMICRO).^[^ ^8^, ^9^, ^10^, ^11^ ^]^ Despite their success, challenges remain in standardizing dECM production, tuning its properties for specific applications, advanced manufacturing, and scaling up for clinical use.^[^ ^12^, ^13^, ^14^ ^]^ In particular, the fabrication of dECM into specialized structures tailored for medical applications remains a significant challenge. The bioactivity of proteins and growth factors can be compromised by standard manufacturing practices, particularly with the use of organic solvents, vigorous mechanical agitation, and heat.^[^ ^15^, ^16^ ^]^ A pressing need exists for manufacturing methods that can fabricate complex dECM geometries while maintaining the regenerative potential of the native tissue. In this perspective, we discuss clinical use of current dECM biomaterials and advances in the fabrication of dECM biomaterials that can broaden applications. Finally, we overview the challenges that current and future dECM biomaterials face in their path to clinical translation.

## 2 From Nature to the Clinic: ECM Biomaterials in Regenerative Medicine

**Figure 1:**
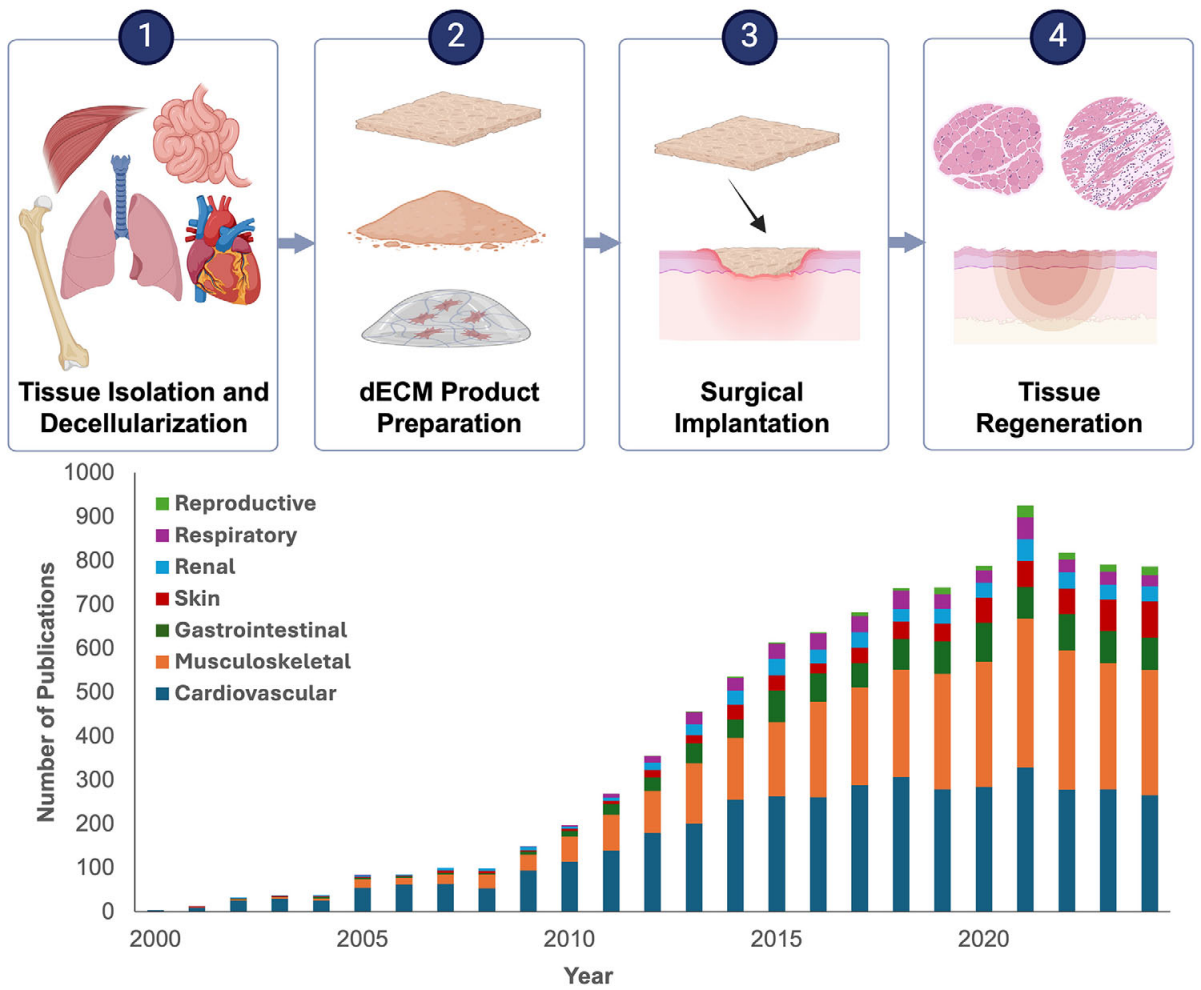
Schematic of dECM biomaterials used to promote tissue regeneration. Following tissue isolation and decellularization, dECM products are prepared and implanted surgically in various soft tissue applications to promote site‐specific tissue regeneration. Publication trends of decellularized tissue since 2000 highlight the growing research interest in ECM biomaterials and the expanding clinical applications. PubMed published article results for “Decellularized Tissue” are provided by year since 2000, broken down by organ system application: cardiovascular (heart, blood vessel, vascular), musculoskeletal (muscle, bone, tendon, cartilage), gastrointestinal (liver, intestine, stomach), skin, renal (kidney, bladder), respiratory (lungs), and reproductive (ovary, uterus). A total of 7266 published articles were used to develop the presented graph. Created in BioRender. Laude, M. (2025) https://BioRender.com/nm2fdu7.

Within each tissue or organ, the extracellular matrix exists in a state of dynamic reciprocity with the resident cell population.^[^ ^17^ ^]^ In addition to providing structural support from collagen, proteoglycans, fibronectin, laminin, elastin, and other glycoproteins, the ECM also contains a wealth of growth factors, extracellular matrix vesicles, and signaling molecules that play critical roles in regulating and maintaining tissue homeostasis, growth, differentiation, vascularization, and maturation.^[^ ^17^, ^18^, ^19^, ^20^ ^]^ Successful clinical translation of ECM biomaterials has relied on these intrinsic biological properties that support tissue repair, regeneration, and functional restoration.^[^ ^21^, ^22^ ^]^ Initially inspired by the biological importance of ECM in tissue structure and function, early studies focused on isolating and characterizing ECM components such as collagen, elastin, and glycosaminoglycans.^[^ ^23^, ^24^, ^25^, ^26^ ^]^ Although single component scaffolds (e.g., collagen, elastin) have great utility in a wide range of clinical applications, the motivation for using more complex ECM biomaterials lies in their ability to mimic the native cellular microenvironment and provide a myriad of biochemical cues to orchestrate remodeling and regeneration. Decellularized tissue, which removes cellular and antigenic content from tissues while preserving ECM physiochemical features, has emerged as a promising scaffold for tissue engineering, **Figure** **1**. Depending on the source tissue and method of decellularization, dECM scaffolds may retain native biochemical and physical cues that guide cell behavior. Upon implantation, dECM‐based scaffolds can promote a process termed “constructive remodeling” or the de novo formation of site‐appropriate, functional tissue.^[^ ^14^ ^]^ As part of this process, dECM degradation products act as chemotactic signals that attract reparative immune cells and promote a pro‐reparative, remodeling‐supportive macrophage phenotype.^[^ ^27^, ^28^, ^29^, ^30^, ^31^ ^]^ Although the precise mechanisms remain unclear, early proregenerative macrophage polarization has been linked to enhanced tissue healing and constructive remodeling, contributing to the clinical success of many marketed dECM‐based products.^[^ ^14^, ^32^ ^]^ These native bioinstructive properties of dECM biomaterials and the corollary regenerative potential is unsurpassed by current synthetic approaches. These properties have made dECM biomaterials indispensable in regenerative medicine, offering promising solutions for tissue repair, organ reconstruction, and disease modeling. As shown in **Figure** 1, there has been exponential interest in dECM as a regenerative biomaterial for a wide range of clinical applications over the last 25 years.

The development of dECM biomaterials has been shaped by decades of interdisciplinary research spanning biology, materials science, and clinical applications. In clinical practice, dECM‐based biomaterials were first used as patches or sheets for surgical applications, with the most common clinical use being in soft tissue applications such as hernia repair, breast reconstruction, and chronic wound treatment, **Table** **1**. Current efforts are expanding dECM biomaterial use into areas like peripheral nerve repair, dental bone augmentation, and ischemic heart injury.^[^ ^14^, ^19^ ^]^ For example, commercial products like MatriStem and GraftJacket are employed for wound healing, hernia repair, and reconstructive surgeries. These scaffolds may provide structural integrity and promote cell infiltration and angiogenesis. Decellularized heart valves and blood vessels (e.g., CryoValve, PhotoFix, Tutopatch) and dECM cardiac patches (e.g., CorMatrix) are used in repair or replacement of heart valves, large vasculature, and congenital heart defects. In addition, dECM biomaterials (e.g., Surgisis for urological repair) are used in procedures including bladder augmentation and pelvic floor reconstruction. These materials promote site‐specific regeneration while reducing the risks of fibrosis and infection. ECM biomaterials can be further processed into powders or solubilized for injection. Several clinical products utilize this powdered form, including bladder matrix (MicroMatrix) and micronized acellular dermis (Micronized AlloDerm). Enzymatic digestion of particulate dECM has been used to generate an dECM solution that when neutralized and adjusted to physiological pH and ionic strength can self‐assemble into a nanofibrous hydrogel. VentriGel, a porcine myocardial dECM, is being evaluated for clinical safety, feasibility, and preliminary efficacy of percutaneous transendocardial delivery in early and late MI patients with left ventricular dysfunction.^[^ ^33^ ^]^ Over the past 30 years, dECM biomaterials have transitioned from experimental tools to clinically adopted therapies, offering versatile solutions for tissue repair and regeneration. As research continues to refine their bioactivity, structural properties, and delivery methods, dECM scaffolds are poised to play a pivotal role in next‐generation regenerative medicine across a broad range of clinical applications.

**Table 1:** List of dECM biomaterial clinical products including source, manufacturer, application, and regulatory classification. Reproduced with permission.^[^ ^14^ ^]^ Copyright 2024, Advanced Drug Delivery Reviews.

Species	Tissue source	Product	Company	Application	Regulatory classification
Human	Dermis	Alloderm	BioHorizons	Soft tissue	FDA‐HCTP
		GraftJacket	Weith Medical	Soft tissue	FDA‐HCTP
		DermaSpan	Zimmer Biomet	Soft tissue, tendon	FDA‐HCTP
Porcine	Amniotic membrane	AmnioExcel	lntegra LifeSciences	Wound healing	FDA‐HCTP
	Bladder	MatriStem	ACell	Wound healing	FDA‐510(k), EMA‐CE
	Dermis	Permacol	Medtronic	Hernia, wound healing, tendon, soft tissue	FDA‐510(k), EMA‐CE
		Strattice	LifeCell Corporation	Hernia, soft tissue	FDA‐510(k), EMA‐CE
		XCM Biologic Tissue matrix	Ethicon	Hernia, tendon, wound healing	FDA‐510(k), EMA‐CE
		XenMatrix	Davol Inc.	Hernia	FDA‐510(k)
	Liver	MiroMesh	Miromatrix Medical Inc.	Hernia	FDA‐510(k)
	Peritoneum	Meso BioMatrix	DSM	Soft tissue	FDA‐510(k)
	Small intestinal submucosa (SIS)	CorMatrix	CorMatrix Cardiovascular	Cardiovascular	FDA‐510(k), EMA‐CE
		OASIS	Cook Biotech	Wound healing	FDA‐510(k)
		Surgisis	Cook Medical	Hernia, nerve	FDA‐510(k)
Bovine	Ventricle fetal dermis	VentriGel Primatrix	Ventrix lntegra LifeSciences	Cardiovascular Wound healing	FDA‐premarket approval (PMA) (IND) FDA‐510(k), EMA‐CE
		Surgimend	lntegra LifeSciences	Hernia	FDA‐510(k), EMA‐CE
		TissueMend	Stryker	Tendon	FDA‐510(k)
	Mesenteric vein	ProCol	LeMaitre Vascular	Cardiovascular	FDA‐PMA
	Pericardium	Tutomesh	RTI Biologics	Hernia, wound healing, soft tissue	FDA‐510(k)
Ovine	Forestomach	Veritas Endoform	Baxter Healthcare Corporation Hollister Woundcare	Hernia, soft tissue Wound healing	FDA‐510(k), EMA‐CE FDA‐510(k), EMA‐CE
Equin	Bone	Gen‐Os	OsteoBiol Tecnoss	Bone, densitry	EMA‐CE
	Pericardium	Matrix Patch	Autotissue	Cardiovascular	EMA‐CE
Fish (Cod)	Dermis	Kerecis Omega 3 Wound	Kerecis Ltd.	Wound healing	FDA‐510(k), EMA‐CE

## 3 Advances in dECM Biomaterial Manufacturing

**Figure 2:**
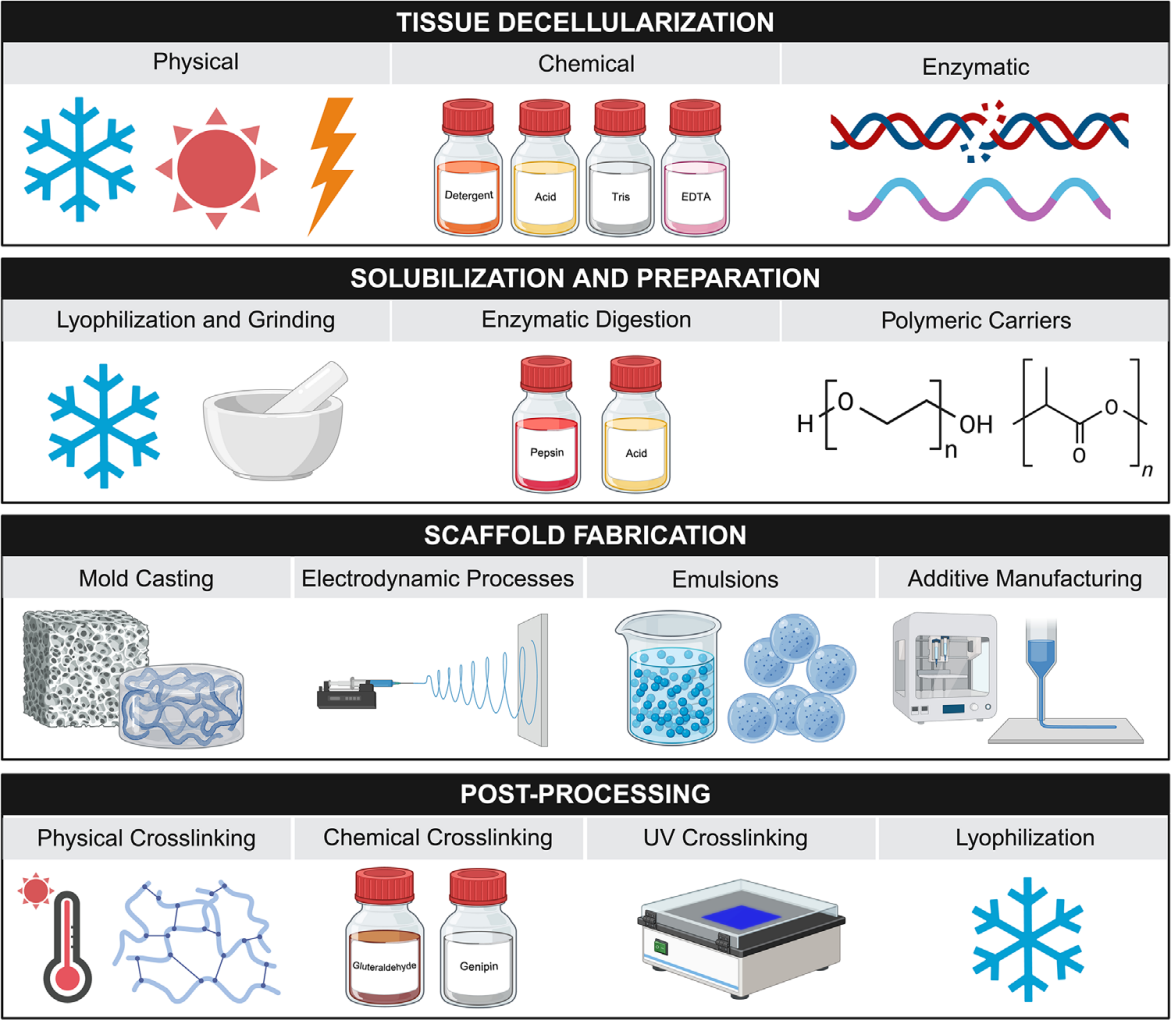
Process of dECM preparation, solubilization, fabrication, and postprocessing. Tissue decellularization involves physical (e.g., freeze–thaw cycles, electroporation, sonication), chemical (e.g., detergents, acids and bases, tris, ethylenediaminetetraacetic acid (EDTA)), and enzymatic (e.g., DNase, RNase, trypsin) methods. After decellularization, dECM is solubilized through lyophilization, grinding, and enzymatic digestion. Synthetic polymers such as polyethylene glycol and polylactic acid have been used as a processing aid for scaffold fabrication. Following dECM solution or suspension preparation, dECM scaffolds are fabricated through mold casting, electrodynamic processes, emulsion‐based fabrication, and additive manufacturing. After fabrication, post‐processing methods including physical, chemical, and UV cross‐linking, and lyophilization are used to further modify the properties of the dECM scaffold. Created in BioRender. Laude, M. (2025) https://BioRender.com/dlq2r14.

Fabricating dECM biomaterials into complex structures remains challenging given that bioactive components can be damaged by traditional processing methods that use high temperatures or organic solvents. As a result, clinical use has mostly been limited to native dECM sheets, ground powders, and solubilized dECM components for injectable formulations.^[^ ^34^ ^]^ The manufacturing of dECM biomaterials has made significant strides in recent years, driven by advancements in material science and manufacturing methodologies. Herein, we provide a general overview of the key processing steps for preparing dECM scaffolds from tissue decellularization, dECM biomaterial processing and preparation, scaffold fabrication, and postprocessing modifications, **Figure** **2**. We have grouped the fabrication techniques based on fabrication principle with the following classifications: mold casting (hydrogels and freeze‐dried foams), electrodynamic processes (electrospinning and electrospraying), emulsion‐based fabrication, and additive manufacturing (dynamic light processing printing and extrusion 3D printing). Summary tables outlining the range of parameters that have been used in each of the manufacturing setups has been included in the Supporting Information.

### 3.1 Tissue Decellularization

Following tissue isolation and removal of unwanted tissue layers, decellularization of the tissue is used to remove cellular and antigenic materials in order to minimize or prevent adverse host responses when the decellularized tissue is implanted. Decellularization is achieved through three primary means: physical, chemical, and enzymatic. Physical methods are meant to disrupt or lyse the cell membranes to release intercellular material and improve the efficacy of subsequent chemical and enzymatic decellularization steps. The most common physical methods are freeze–thaw cycling, sonication, electroporation, vacuum, or mechanical processing.^[^ ^21^, ^35^, ^36^, ^37^ ^]^ Next, chemical methods are used to further solubilize the cell membrane, denature proteins, and deactivate enzymes. The most common chemical agents used are ionic detergents (e.g., sodium dodecyl sulfate, sodium deoxycholate) and nonionic detergents (e.g., Triton X‐100) which facilitate cell debris removal.^[^ ^37^ ^]^ Acids (e.g., peracetic acid, HCl, acetic acid, lactic acid) and bases (e.g., NaOH, NH_4_OH) are used to solubilize cytoplastic contents. Hypo‐ and hypertonic solutions are often used in combination with chemical agents and detergents to induce osmotic shock and induce cell lysis.^[^ ^7^, ^21^, ^36^ ^]^ Finally, enzymatic methods are used to disrupt cell attachment and degrade residual genetic material and proteins. DNase, RNase, and trypsin are the most commonly used enzymatic agents.^[^ ^7^, ^21^, ^36^ ^]^ Current literature relies on a combination of physical, chemical, and enzymatic decellularization to reduce cellular content below recommended levels (< 50 ng mg^−1^ double‐stranded DNA content in ECM; < 200 base pair DNA fragment size) and remove visible nuclear material in tissue stained with DAPI or H&E.^[^ ^7^ ^]^ Although these guidelines are widely used to indicate sufficient decellularization, they do not fully indicate the potential for adverse responses to residual immunological material not removed in the processing.^[^ ^38^, ^39^, ^40^ ^]^ ASTM F3354 provides recommendations for decellularization characterization and the future development of decellularization acceptance criteria, but does not fully define thresholds, demonstrating a clear need for defined standards to improve the standardization of dECM fabrication.^[^ ^41^ ^]^

The reduction of cellular content and prevention of an adverse host response is essential for the preparation of dECM scaffolds, but the decellularization process risks damaging the composition and relative architecture of the ECM, potentially impeding its bioactivity. Selection of an appropriate decellularization method will depend on the selected tissue and intended application requirements. There is a balancing act between the removal of cellular content and disruption of the ECM structure by detergents and enzymatic agents. Damage to the ECM structure can negatively impact structural features and mechanical properties^[^ ^42^, ^43^, ^44^ ^]^ as well as remove or denature desirable biological molecules with the corresponding loss of bioactivity.^[^ ^31^, ^37^, ^44^ ^]^ While most authors comment on the levels of decellularization achieved, whether that be as DNA content of decellularized tissue or the percentage of cell removal, few authors characterize the accompanying changes in dECM bioactivity. Mechanical and biological comparisons between dECM before and after processing should be further explored to fully examine the effects of decellularization. Finally, chemical or enzymatic residues remaining in the tissue can induce an adverse immune response.^[^ ^45^, ^46^ ^]^ The potential cytotoxic or immunological properties of residual agents following decellularization requires further examination to prevent adverse responses to dECM scaffolds.

### 3.2 dECM Solubilization and Preparation

#### 3.2.1 Intact Tissue Processing

After decellularization, dECM can be used in its intact form without additional processing. This has many benefits, primarily the ability to retain the structure and composition of the native tissue. Using dECM fully intact minimizes additional processing steps and limits damage that can come through pulverizing or digesting the tissue. Current clinical dECM products are often used in hydrated or lyophilized sheet form, with lyophilization being used to preserve many commercially available biological materials.^[^ ^34^ ^]^ However, intact dECM cannot be used for most other form factors as electrodynamic processes, casting, emulsions, and 3D printing typically require some level of solubilization and solution or suspension preparation.

#### 3.2.2 Particulate Processing

In preparing solutions or suspensions, the dECM is first lyophilized and ground into a powder in preparation for scaffold fabrication. The grinding of lyophilized dECM is typically done with a cryomill in which the dECM is frozen with liquid nitrogen and pulverized by a metal ball, creating small, uniform dECM particulates. Although the grinding of dECM leads to the loss of the macroscopic native ECM structure, it maintains the microscale composition and organization within the particulate granule. Milled dECM particulates can be made into suspensions for fabrication. Particulate‐based suspensions are used in electrospinning, electrospraying, freeze‐casting, and 3D printing.^[^ ^47^, ^48^, ^49^, ^50^, ^51^, ^52^, ^53^, ^54^, ^55^, ^56^, ^57^, ^58^, ^59^, ^60^, ^61^, ^62^ ^]^ These suspensions are highly heterogeneous and often require polymeric additives as a processing aid to achieve requisite rheological properties for scaffold fabrication.^[^ ^47^, ^50^, ^51^, ^52^, ^53^, ^54^, ^55^, ^60^, ^61^, ^62^, ^63^ ^]^ However, the addition of polymeric additives reduces the dECM concentration and can impede cell–dECM interactions. The reduction of dECM exposure to the surrounding microenvironment can result in delayed delivery of bioactive cues. If the particulates are fully encapsulated in the synthetic polymer, the interaction between cells and the dECM will not occur until the polymer has degraded. The inclusion of synthetic polymers can also lead to an adverse host response.^[^ ^64^, ^65^, ^66^, ^67^ ^]^ Thus, milled dECM may require further processing to achieve appropriate rheological properties for fabrication without polymeric additives.

#### 3.2.3 Solubilization via Digestion

After decellularization, lyophilization, and milling, the dECM is often digested to improve solubility for solution‐based processing. The dECM powder is enzymatically digested to break down the dECM components into a more homogenous solution with better rheological properties for fabrication. Enzymatic digestion can be used to prepare solutions for electrodynamic processes, freeze‐casting, and 3D printing, and is used invariably to prepare solutions for hydrogels and emulsion‐based systems. Pepsin is the most used enzyme for dECM digestion.^[^ ^68^ ^]^ It is used in an acidic solution, typically either acetic acid or hydrochloric acid. The acidic environment activates the pepsin and can help denature proteins, enhancing the enzymatic activity of pepsin.^[^ ^69^ ^]^ There are three established solubilization protocols used for these gel precursor solutions: Freytes, Voytik–Harbin, and Uriel methods.^[^ ^70^, ^71^, ^72^ ^]^ The Freytes method combines milled dECM with pepsin and hydrochloric acid over 48 h.^[^ ^70^ ^]^ Similarly, the Voytik–Harbin method adds milled dECM to pepsin and acetic acid.^[^ ^71^ ^]^ The Uriel method requires the homogenization of the milled dECM in dispase, extraction of ECM proteins in urea, and centrifugation to remove nonsoluble ECM components.^[^ ^72^ ^]^ Digestion is generally considered complete when no visible dECM particles remain, and the dECM digest is usually filtered prior to subsequent processing. The dECM digest can be used as an acidic pregel, typically for casting or emulsion processes. For 3D printing, sodium hydroxide and phosphate buffered saline are typically used to neutralize the dECM digest and adjust the salt concentration prior to fabrication. For electrodynamic processes, the dECM digest is often lyophilized again before being added to a new solvent.

Although effective in modulating rheological properties to facilitate fabrication, the use of enzymes in dECM preparation has the potential to decrease the bioactivity of proteins and growth factors that provide dECM with its regenerative potential. Pepsin works by cleaving peptide bonds in protein molecules, effectively breaking down collagen, fibronectin, laminin, elastin, and many other proteins found in the extracellular matrix into smaller peptide fragments.^[^ ^73^ ^]^ Beyond the decrease in protein bioactivity, the use of pepsin can also reduce the mechanical properties of the resulting dECM scaffold.^[^ ^74^ ^]^ Collagen, the most abundant protein in ECM, plays an essential role in providing ECM with tensile strength and structural support.^[^ ^75^ ^]^ The cleavage of collagen during pepsin digestion reduces the molecular weight of collagen, resulting in a reduction in mechanical properties.^[^ ^76^ ^]^ Although enzymatic digestion is widely used and heavily relied upon to fully solubilize dECM components prior to fabrication, researchers must balance the benefits to processing with the potential loss of the regenerative potential of fabricated scaffolds.^[^ ^74^, ^77^ ^]^

### 3.3 dECM Scaffold Fabrication Methods

#### 3.3.1 Mold Casting

One of the least complex fabrication methods of dECM fabrication is the casting of hydrogels or freeze‐dried foams into molds. First, dECM hydrogels are cross‐linked, water‐swollen 3D network structures that mimic native tissue structure with adjustable mechanical properties that can influence cell growth and proliferation.^[^ ^78^, ^79^, ^80^ ^]^ dECM hydrogels have been prepared from a wide variety of tissue sources and species, as summarized in **Table** S1 (Supporting Information). However, regardless of tissue source or species, all dECM hydrogels rely on digestion to fully solubilize the ECM components and develop pregel solutions with adequate rheological properties for casting.

As previously detailed, digestion with enzymes such as pepsin can result in significant degradation and denaturation of ECM proteins, reducing the bioactivity and regenerative potential of dECM hydrogel scaffolds. Hussey et al. evaluated a novel method of solubilization using ultrasonic cavitation that uses a sonicator horn to fully solubilize dECM powder suspended in a saline solution.^[^ ^81^ ^]^ Beyond retaining protein structure and bioactivity, ultrasonic cavitation has additional benefits. The solubilization process occurred fully between 30 and 500 s with concentrations of dECM between 25 and 100 mg mL^−1^. Comparatively, pepsin digestion can take 24–72 h to achieve full solubilization and has limited concentration limits of typically up to 20 mg mL^−1^ to maintain adequate rheological properties needed for gelation. Thus, ultrasonic cavitation provides the potential for scale‐up needed for clinical application that is not achievable with pepsin‐digested solutions. A wide range of dECM concentrations have been used in hydrogel fabrication, with 10–20 mg mL^−1^ being the most common.^[^ ^82^, ^83^, ^84^, ^85^, ^86^, ^87^, ^88^, ^89^, ^90^ ^]^ The prepared pregel solution can be injected between plates or cast in molds before cross‐linking. The primary method of cross‐linking used in hydrogel casting is heat gelation.^[^ ^82^, ^85^, ^86^, ^87^, ^88^, ^89^, ^90^, ^91^, ^92^, ^93^, ^94^ ^]^ Incubation of the pregel solution in a 37 °C incubator leads to gelation primarily due to the self‐assembly of fiber‐like aggregation of collagen, a process driven by the exclusion of water and increased hydrophobic interactions between collagen molecules.^[^ ^95^ ^]^ However, these physical gels can be unstable, so chemical cross‐linking after gelation (e.g., glutaraldehyde) is commonly used to increase stiffness and retard degradation.^[^ ^96^ ^]^ Beyond chemical cross‐linking agents, functionalization of dECM proteins with methacrylate groups has been used to enable UV cross‐linking.^[^ ^97^, ^98^ ^]^ Methacrylation reduces the introduction of potentially cytotoxic agents while enabling the stabilization and enhancement of mechanical properties of dECM hydrogels. In summary, dECM hydrogels are a widely explored platform used for tissue repair and regeneration across a range of bioengineering applications. Moving forward, renewed efforts to transition away from pepsin digestion are necessary for improving the fine‐tuning of mechanical properties such as gelation kinetics, stiffness, and degradation profiles based on application. The development of solubilization methods beyond digestion will also expand the scale‐up potential, improving the suitability of dECM hydrogels for clinical translation.

In addition to hydrogels, researchers have developed methods to generate porous dECM scaffolds that can promote cellular infiltration and viability. The development of dECM foams follows two primary methods: casting milled, digested dECM solution into a mold to produce foams or casting electrosprayed microparticles into a mold to produce bead foams.^[^ ^57^, ^58^, ^59^, ^99^, ^100^, ^101^, ^102^, ^103^ ^]^ Both processes use a concentration range of 20–100 mg mL^−1^ of dECM in solution and generate the final foam structure using a final lyophilization or freeze‐drying step.^[^ ^57^, ^58^, ^99^, ^100^, ^101^, ^102^ ^]^ The milled, digested dECM solution is frozen in molds prior to lyophilization, allowing ice crystals to form throughout the sample, which are subsequently sublimated under vacuum, resulting in foams with relatively uniform, interconnected pores. Densification of the dECM during lyophilization allows for inter‐ and intramolecular bonds to form among ECM molecules (e.g., hydrogen bonding, electrostatic interactions, hydrophobic interactions) that stabilize the scaffold structure. These freeze‐dried dECM foams have tunable porosity and pore size, which is important for cellular infiltration and can impact many biological processes needed for regeneration including matrix deposition and angiogenesis. Pore size is determined by the freezing temperature and rate, with higher temperatures resulting in larger ice crystals and therefore larger pores.^[^ ^104^ ^]^ For the fabrication of bead foams, pore size is controlled by the size of the electrosprayed microparticles, providing much more precise control than freeze rate alone.^[^ ^57^, ^99^ ^]^ A summary of different tissue sources and setups that have been used for casting of dECM foams is available in **Table** S2 (Supporting Information). Although polymeric additives such as silk fibroin have been used in dECM foam solutions, they are not required for successful foam fabrication of either milled or bead foams.^[^ ^103^ ^]^ These fabrication methods have been used on a limited number of tissue sources with human and porcine adipose tissue comprising the majority of dECM foams.^[^ ^57^, ^58^, ^59^, ^99^, ^101^, ^102^ ^]^ Porcine heart and human placenta have also been used to make freeze‐dried foams, but further exploration is needed to expand this application to additional tissue sources.^[^ ^100^, ^103^ ^]^ The primary limitation in the fabrication of freeze‐dried dECM foams is the reliance on enzymatic digestion. Established protocols use enzymatic digestion to fully solubilize the dECM prior to casting, resulting in protein degradation and denaturation that can affect the native bioactivity of the dECM. Mendibil et al. examined a new method of dECM solubilization using acetic acid. By allowing decellularized porcine cartilage to sit in 0.5 m acetic acid for 24 h prior to lyophilization, full solubilization of the dECM was achieved.^[^ ^105^ ^]^ Although acetic acid can lead to the denaturation of ECM proteins such as collagen, it does not cause proteolytic cleavage of ECM proteins. The use of acetic acid as a solubilization agent provides a significant improvement in the maintenance of dECM bioactivity and should be further investigated in scaffold preparation and fabrication. Overall, the development of freeze‐cast dECM scaffolds provides a porous architecture that is often desirable in tissue engineering applications. Moving forward, dECM freeze‐casting can be advanced to improve the tunability of mechanical properties, degradation rates, and structural consistency. The current reliance on enzymatic digestion must be addressed to improve foam mechanics. Additionally, enhanced control over pore architecture and the incorporation of cross‐linking methods should be explored to achieve dECM foam scaffolds with functional reliability and broader therapeutic potential.

#### 3.3.2 Electrodynamic Processing

Electrodynamic processing of biomaterials such as electrospinning and electrospraying utilizes an electrostatic field to draw polymeric solutions to form nonwoven fiber meshes or microparticles. Electrospinning has been utilized to make fibrous dECM scaffolds given its control of the resulting fiber diameter and microarchitecture.^[^ ^106^, ^107^ ^]^ There are two types of dECM solutions used in electrospinning. The first is enzyme‐digested dECM which can be spun with or without a polymer carrier.^[^ ^108^, ^109^, ^110^, ^111^, ^112^ ^]^ The digestion of dECM to its base components allows for macromolecular entanglement that can withstand the electrostatic forces of electrospinning to form continuous fibers. The second method incorporates particulate dECM into a polymer carrier solution using either natural or synthetic polymers.^[^ ^47^, ^50^, ^51^, ^52^, ^53^, ^54^, ^55^, ^63^, ^113^ ^]^ The nondigested dECM particles require the polymeric solution to impart sufficient elasticity to achieve continuous fiber formation. A summary of different tissue sources and setups that have been used for electrospinning of dECM scaffolds is available in **Table** S3 (Supporting Information). The most commonly used solvent for preparing electrospinning solutions is hexafluoroisopropanol (HFIP). Solution electrospinning of pepsin‐digested dECM can be achieved with a variety of tissue sources and polymer carriers. Porcine is the most common species from which tissue is isolated and decellularized for electrospinning, and porcine skeletal muscle, cartilage, and small intestinal submucosa have been successfully electrospun.^[^ ^109^, ^110^, ^111^, ^112^ ^]^ Human and bovine tissues have also been used.^[^ ^108^, ^114^ ^]^ After fabrication, chemical cross‐linking methods including glutaraldehyde and genipin are commonly used to further stabilize the electrospun mesh.^[^ ^47^ ^–^ ^49^, ^114^ ^]^ UV cross‐linking has also been used with methacrylated dECM.^[^ ^109^ ^]^ Synthetic polymers including polycaprolactone (PCL), poly(lactic‐*co*‐glycolic acid) (PLGA), and polyurethanes have been used with the dECM digest to make blended polymer fiber scaffolds.^[^ ^108^, ^110^, ^111^, ^112^ ^]^ The dECM content is relatively limited in these blends with most meshes having a dECM content of 10% w/v or lower in the spinning solution. Solution electrospinning of scaffolds with particulate dECM has primarily used porcine tissue, but rabbit, human, and rat tissue have also been electrospun.^[^ ^47^ ^–^ ^49^, ^54^ ^]^ The tissue sources used include adipose, skeletal muscle, lung, pancreas, meniscus, cartilage, tendon, and brain. PCL is the most commonly used polymer carrier,^[^ ^52^, ^53^, ^63^, ^113^ ^]^ but silk fibroin, gelatin, poly‐l‐lactic acid, and polyhydroxybutyrate have also been used.^[^ ^47^, ^50^, ^51^, ^54^, ^55^, ^63^ ^]^ Similarly to dECM digest blends, the dECM loading content in these composite meshes is typically 10% w/v or lower in the solution. Different ratios of polymer to dECM have been explored, with Gao et al. electrospinning 6% w/w decellularized porcine meniscus and 8% w/w PCL in ratios of 0:5, 1:4, 2:3, 3:2, 4:1, and 5:0.^[^ ^113^ ^]^ As previously mentioned, the use of polymercarriers in scaffold fabrication can limit dECM content, resulting in low cell–dECM interactions. To address this limitation, Smoak et al. electrospun decellularized rabbit skeletal muscle using a new suspension electrospinning method without a polymer carrier.^[^ ^48^ ^]^ Ground, undigested, decellularized muscle was suspended in HFIP and allowed to stir overnight prior to electrospinning. After electrospinning, glutaraldehyde was used to chemically cross‐link and stabilize the fiber scaffolds. Building on this work, Jones et al. successfully electrospun porcine small intestinal submucosa (SIS) suspensions in HFIP without a polymeric carrier or enzymatic digestion.^[^ ^56^ ^]^ Given that these suspensions have reduced macromolecular interactions compared to fully solubilized dECM digests, a physical homogenization step is critical to achieve sufficient solution elasticity to form continuous fibers. These meshes were shown to maintain similar bioactivity including immunoregulatory behavior and angiogenesis compared to dECM that has not been electrospun.^[^ ^56^ ^]^ This bioactivity retention was attributed to the reduced processing and solubilization of the suspension as compared to traditional dECM digest solutions. One significant limitation in dECM electrospinning to date is the reliance on HFIP and other organic solvents given potential residual toxicity and environmental hazards that pose a significant barrier to industrial scale‐up and clinical translation. Baiguera et al. successfully electrospun decellularized rat brain with a gelatin carrier using 9:1 ratio of acetic acid and DI water as the solvent.^[^ ^47^ ^]^ The decellularized dECM was added in particulate form to the acetic acid/DI water mixture and ultrasonicated before the addition of gelatin. Following electrospinning, the meshes were soaked in genipin to facilitate cross‐linking. Although this process successfully limited the use of harsh solvents and enzymatic digestion, only 1% w/w dECM powder with respect to gelatin was loaded into the suspension. This is likely because acetic acid is not as effective of a solvent for dECM, thereby limiting the loading limits in suspension. Overall, electrospinning provides a viable method to fabricate dECM into fiber‐based scaffolds with tunable architecture, but the current reliance on enzymatic digestion, organic solvents, and polymer carriers may limit the regenerative potential of the resulting meshes. These limitations highlight the need for innovative fabrication and suspension preparation strategies that preserve the bioactivity of dECM while enabling precise control over mesh architecture.

Electrospraying of microparticles follows similar principles to electrospinning, using an electrostatic drawing force to generate discrete microparticles from a polymer solution rather than continuous fibers. To prepare dECM for electrospraying, it is typically first milled to create fine particles and digested with pepsin and an acetic acid solvent. The digested dECM is then suspended in acetic acid at concentrations ranging from 15 to 200 mg mL^−1^ and commonly homogenized before being electrosprayed into a liquid nitrogen receptacle.^[^ ^57^, ^101^, ^102^, ^115^ ^]^ The microparticles can then be collected and lyophilized to generate individual particles or packed into a mold to achieve a porous foam through freeze casting.^[^ ^99^, ^101^, ^115^ ^]^ Similar to electrospinning, the strength of the electrostatic field, distance to the collector, and solution flow rate allow for precise control over microparticle size. Electrosprayed microparticles do not require a polymer carrier for fabrication, but collagen has been used as an additive.^[^ ^99^ ^]^ A summary of different tissue sources and setups that have been used for electrospraying of dECM microparticles is available in **Table** S4 (Supporting Information). Relatively few tissue sources have been used for this technique, with primarily human and porcine adipose tissue having been successfully electrosprayed.^[^ ^57^, ^99^, ^101^, ^102^ ^]^ Kornmuller and Flynn electrosprayed decellularized porcine dermis and heart, exhibiting the potential to apply this fabrication technique to other tissue sources, but more work is needed to explore whether electrospraying can be universally applied across tissue sources.^[^ ^115^ ^]^ Currently, one of the biggest limitations for fabricating electrosprayed microparticles is the reliance on enzymatic digestion prior to fabrication. Morissette Martin et al. successfully electrosprayed decellularized human adipose tissue in particulate form.^[^ ^57^ ^]^ The decellularized adipose was milled and suspended in acetic acid prior to electrospraying into liquid nitrogen. These particulate microparticles showed proangiogenic and immunomodulatory effects when cultured with adipose‐derived stem cells. This work introduces a promising new method of fabricating electrosprayed particles without the use of enzymes or polymer carriers that could negatively impact the regenerative potential. Electrospraying dECM is a versatile fabrication method that can result in individual microparticles or sintered porous foams, but it has not been significantly explored, and there are some concerns regarding throughput. The expansion of this fabrication technique to additional sources is an important next step to demonstrate technique versatility and to examine the effect of tissue source on dECM microparticle bioactivity and function.

#### 3.3.3 Emulsion‐Based Fabrication

Emulsions are mixtures of two or more immiscible liquids with the dispersion of one liquid as droplets in the second, continuous phase. For dECM emulsions, the droplet phase is typically an aqueous dECM solution that is dispersed in an oil‐based continuous phase. This process is generally used to generate dECM microparticles from the droplet phase, either through bulk emulsion or microfluidic setups. Microfluidic emulsion systems feed the aqueous phase and continuous phase from two separate channels into one channel, with the size of the droplets and subsequent microparticles controlled by the flow rates and rheological properties of the liquids. A summary of different tissue sources and setups that have been used for emulsion‐based fabrication of dECM is available in **Table** S5 (Supporting Information). Typically, the concentration of dECM digest solution is low, between 1% and 4% w/v.^[^ ^116^, ^117^, ^118^, ^119^, ^120^, ^121^ ^]^ Similar preparation to dECM hydrogel solutions is used with pepsin digestion in an acid, either hydrochloric acid or acetic acid,^[^ ^116^, ^117^, ^118^, ^119^, ^120^, ^121^ ^]^ with sodium hydroxide often used to neutralize the acidic pregel solution and buffer to adjust the salt concentration. The continuous phase can be a variety of hydrophobic liquids including mineral oil, polyvinyl alcohol, paraffin oil, gelatin, and olive oil. After fabrication, heat gelation is most commonly used to physically cross‐link the final microparticles.^[^ ^116^, ^122^, ^123^ ^]^ Photoinitiated cross‐linking and glutaraldehyde treatment have also been used as chemical cross‐linking methods.^[^ ^117^, ^121^ ^]^ Luo et al. used solvent extraction and lyophilization after fabrication to create highly porous microspheres.^[^ ^124^ ^]^ Emulsion‐fabricated microparticles have been shown to maintain similar topography to hydrogels of the same composition but with a higher elastic modulus.^[^ ^123^ ^]^ Polymeric additives including gelatin, hyaluronic acid, alginate, and PLGA are often added to the aqueous solution to adjust solution viscosity.^[^ ^117^, ^118^, ^119^, ^120^, ^121^, ^122^, ^124^ ^]^ Wang et al. utilized a droplet‐based system to fabricate solid dECM microparticles without a polymer carrier.^[^ ^123^ ^]^ The aqueous phase contained decellularized porcine ventricle digested with pepsin and dissolved in a digestion buffer, and the continuous phase contained olive oil with a surfactant. Lin et al. had similar success fabricating solid dECM microparticles using a microfluidic system.^[^ ^116^ ^]^ Decellularized porcine nerves were digested with pepsin in a hydrochloric acid solution, then neutralized in sodium hydroxide and phosphate‐buffered saline (PBS) to form the pregel solution. This solution was fed into the same channel as a mineral oil continuous phase with a surfactant, producing spherical microparticles that were then immersed in a 37 °C water bath for heat gelation. The fabrication of microparticles without polymeric additives improves the delivery potential of these microparticles as the exposure of dECM to the surrounding microenvironment is enhanced. Microparticles can also be cast into molds to form sintered bead foams as described above. Chen et al. lyophilized PLGA, gelatin, and dECM microspheres in a mold before dissolving the gelatin to create a porous structure similar to the bead foams.^[^ ^122^ ^]^ Emulsion‐based systems have been used to fabricate dECM scaffolds and microparticles from a wide variety of tissue sources including porcine nerves, ventricles, cartilage, and skeletal muscle; rat bone; and human adipose tissue, exhibiting the versatility of this fabrication technique. Overall, emulsion‐based fabrication is a promising, tunable platform for the fabrication of dECM microparticles and bead‐sintered foams. Future efforts should focus on tailoring these microparticles for therapeutic applications through the incorporation of cells, antibiotics, and other bioactive components. However, the reliance on digestion must be addressed to optimize particle composition and degradation and adapt dECM microparticles based on clinical needs.

#### 3.3.4 Additive Manufacturing

Two of the most common additive manufacturing methods used for fabricating dECM scaffold are digital light processing (DLP) and extrusion‐based 3D printing. DLP printing is a vat polymerization process, similar to stereolithography, that uses a digital projector for in situ curing of a liquid polymer resin into solid 3D structures layer by layer, with the build platform raising between each exposure to allow recoating.^[^ ^125^, ^126^ ^]^ DLP enables rapid fabrication of complex and precise 3D structures; however, viscosity of the photocurable polymer must be carefully adjusted to achieve quality prints. A summary of different tissue sources and setups that have been used for DLP printing of dECM is available in **Table** S6 (Supporting Information). Most typically, gelatin methacrylate (GelMA) is used as the base photocurable ink with dECM digest added to increase regenerative potential of the construct.^[^ ^125^, ^127^, ^128^, ^129^ ^]^ Due to the viscosity limitations of DLP printing, the loading content of dECM is relatively low (<5% w/v).^[^ ^126^ ^]^ As an alternative to GelMA, Kim et al. used a ruthenium/sodium persulfate (Ru/SPS) photoinitiator with porcine heart and bovine cornea to develop a dECM ink that can achieve a cross‐linked network with visible light exposure.^[^ ^130^ ^]^ The Ru/SPS targeted the tyrosine‐rich protein in the dECM to cross‐link the matrix via formation of dityrosine bonds. Elomaa et al. demonstrated 3D printing of rat liver at higher concentrations of 70 wt% through the direct functionalization of the dECM with methacrylate groups.^[^ ^131^ ^]^ This ink was then printed in a composite resin with methacrylated PCL. Moving forward, developing new functionalized dECM inks and utilizing the native chemistry of the ECM to enable photoinitiated cross‐linking without polymeric additives could expand the utility of DLP in 3D printing dECM scaffolds.

Extrusion 3D printing uses a force, typically pneumatic or piston‐driven, to deposit the ink into a preset orientation. The dECM inks developed for extrusion printing typically have relatively high low‐shear viscosity and are often shear thinning.^[^ ^132^ ^]^ Most commonly, dECM inks used in extrusion printing contain dECM digest, but more recent studies have investigated dECM particle composites. A summary of different tissue sources and setups that have been used for extrusion 3D printing of dECM scaffolds is available in **Table** S7 (Supporting Information). Due to the high viscosities possible for inks used in extrusion printing, dECM can be loaded at much higher weight fractions than possible in DLP, up to 20% w/v. Most inks also use natural and synthetic polymer carriers such as alginate, collagen, PCL, and poly(ethylene glycol) diacrylate (PEG‐DA) to achieve rheological properties needed for extrusion printing. In addition, a wide variety of cells have been added to dECM bioinks including human adipose‐derived stem cells (hASCs), myoblasts, cardiomyocytes, and cardiac progenitor cells.^[^ ^133^, ^134^, ^135^, ^136^, ^137^, ^138^ ^]^ After printing, various cross‐linking methods are used to stabilize the scaffolds including heat gelation and chemical cross‐linking of either the functionalized dECM or the polymer carrier (e.g., GelMA).^[^ ^49^, ^60^, ^62^, ^133^, ^134^, ^135^, ^136^, ^138^, ^139^, ^140^ ^]^ Alternatively, calcium chloride can be used to induce cross‐linking when alginate is used as a polymer carrier.^[^ ^141^, ^142^ ^]^ As with many other fabrication methods, the use of pepsin digestion is common and can have negative effects on scaffold bioactivity. As an alternative, Barthold et al. developed particulate‐based dECM inks.^[^ ^62^ ^]^ Decellularized porcine cartilage was lyophilized and pulverized before being added to Dulbecco's Phosphate Buffered Saline (DPBS) with thiolated hyaluronic acid as a polymer carrier at 20% w/v. The free thiol groups present in the dECM microparticles reacted with the thiolated hyaluronic acid for polymerization which was accelerated at 37 °C. Jang et al. developed a two‐step process to print decellularized porcine cardiac tissue without a polymer carrier.^[^ ^143^ ^]^ Vitamin B‐2, a natural photoinitiator, was added to the dECM as well as cardiac cells isolated from human cardiac tissue. After printing, photo‐cross‐linking was induced with UV irradiation, and physical cross‐linking of the dECM was achieved with incubation at 37 °C. In summary, extrusion‐based 3D printing of dECM provides a highly scalable and tunable system for fabricating complex bioactive scaffolds. An expansion to multinozzle extrusion systems and coaxial printing techniques will enable the creation of complex, heterogeneous architectures specifically tuned based on the application. Additionally, the development of novel dECM bioinks with cross‐linking strategies that reduce the reliance on polymer carriers for printability will expand the capabilities of dECM‐based bioprinted scaffolds and their translation into clinically relevant applications.

### 3.4 Postprocessing of dECM Scaffolds

To lock in the geometry and microarchitecture of dECM scaffolds after fabrication, physical and chemical methods of cross‐linking are used. Most commonly, heat gelation is used to induce physical cross‐linking of cast hydrogels, emulsion‐based microparticles, and 3D printed scaffolds.^[^ ^60^, ^62^, ^82^, ^85^, ^86^, ^87^, ^88^, ^89^, ^90^, ^91^, ^92^, ^93^, ^94^, ^116^, ^122^, ^123^, ^134^, ^135^, ^136^, ^138^ ^]^ As described above, incubation at 37 °C leads to gelation primarily due to the self‐assembly of fiber‐like aggregation of collagen.^[^ ^95^ ^]^ The incubation period can last from a couple minutes to 4 h and is complete when scaffolds have solidified. Additional chemical cross‐linking can be used in conjunction with physical cross‐linking to enhance the stability and mechanical properties of hydrogels, microparticles, and 3D printed scaffolds.^[^ ^96^ ^]^ Formaldehyde and glutaraldehyde are common chemical cross‐linking agents used for the stabilization of dECM scaffolds.^[^ ^84^, ^144^, ^145^ ^]^ Both of these agents form chemical bonds between proteins, forming covalent bonds in a stable cross‐linked network. Although formaldehyde and glutaraldehyde effectively stabilize the structure of dECM scaffolds, each has been shown to have high cytotoxicity and potential for inflammatory response.^[^ ^83^ ^]^ More recently, natural plant‐derived cross‐linking agents have been applied to dECM biomaterials; Výborný et al. explored the use of genipin and *N*‐(3‐dimethylaminopropyl)‐*N′*‐ethyl carbodiimide hydrochloride as dECM cross‐linking agents and showed reduced cytotoxicity as well as improved structural stability and a reduction in enzymatic degradation rates.^[^ ^83^ ^]^ Ma et al. had similar success using procyanidins as a cross‐linking agent to achieve stable mechanical properties and in vitro enzymatic degradation resistance without negatively impacting cytocompatibility.^[^ ^146^ ^]^ Further examination of cross‐linking methods that can balance stability, maintain ECM native structure and biological cues, and prevent an adverse host response is an important future direction. Photoinitiated cross‐linking is another form of chemical cross‐linking that uses UV light to activate photoinitiators or photoreactive groups, causing the formation of covalent bonds between certain chemical groups.^[^ ^147^ ^]^ This method of cross‐linking is often used to stabilize casted hydrogels, emulsion microparticles, and 3D printed scaffolds.^[^ ^97^, ^98^, ^117^, ^125^, ^126^, ^139^, ^143^ ^]^ The two primary methods of introducing photoreactive groups into these scaffolds are through the functionalization of dECM or the inclusion of a photosensitive polymer additive. The dECM can be functionalized with acrylate or methacrylate groups which are polymerized by UV light to form a cross‐linked network.^[^ ^97^, ^98^, ^109^ ^]^ More commonly, these acrylate groups are introduced through polymeric additives such as GelMA or PEG‐DA.^[^ ^117^, ^125^, ^126^ ^]^ Postprocessing cross‐linking plays an important role in enhancing the stability and structural integrity of dECM scaffolds after fabrication. Although physical cross‐linking offers a reversible approach that preserves bioactivity, chemical cross‐linking provides more robust and durable stabilization and mechanical strength, albeit with potentially cytotoxic agents that can induce a chronic foreign body response. Physical and chemical cross‐linking methods – either used independently or in combination – allow for tailored modification of dECM scaffolds to meet functional demands of tissue engineering applications.

Overall, advances in dECM fabrication methods and the potential for hybrid fabrication with synthetic materials allow for the manufacturing of highly customizable dECM scaffolds with a broad range of architectures and geometries. These advances in manufacturing will substantially broaden the potential clinical applications of dECM biomaterials. However, the general reliance on enzymatic digestion during the solubilization of dECM in each fabrication method can have negative effects on the bioactivity and structural stability of the processed dECM. To address these challenges, future research should focus on establishing standardized decellularization protocols and exploring digestion‐free fabrication methods to enhance the bioactivity and structural integrity of dECM scaffolds. Overcoming these limitations will make it possible to increase the regenerative potential of dECM scaffolds for tissue engineering and regenerative medicine.

## 4 Challenges in Translation of dECM Biomaterials in Regenerative Medicine

**Figure 3:**
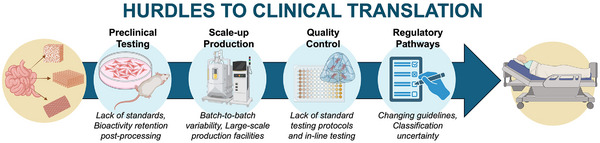
Schematic of challenges in the translational pathway of ECM biomaterials. Extensive preclinical testing is necessary to assess product safety and efficacy, with the subsequent production scale‐up requiring advanced bioreactor systems and comprehensive controls to ensure consistent quality and reproducibility across batches. Regulatory pathways established by governmental agencies must be followed across these steps to achieve consistency and standardization and receive approval for clinical use. Created in BioRender. Laude, M. (2025) https://BioRender.com/yq76247.

As manufacturing advances are poised to broaden clinical applications of dECM biomaterials, there remain several challenges to clinical translation of these technologies. One key concern is the inherent biological variability between tissue sources and donors. Variations in tissue composition and quality can lead to differences in decellularization efficacy and fabrication outcomes, resulting in batch inconsistencies and complicating the standardization of dECM biomaterials for clinical use. New strategies and protocols are needed to address the unique challenges of advancing dECM products through preclinical testing and scalable manufacturing, while ensuring appropriate quality control and navigating regulatory pathways to achieve clinical adoption, **Figure** **3**.

Mammalian tissues have inherent compositional variability, dependent upon the source animal's age and gender, the environmental milieu the source animal has experienced, and the source tissue type. Some degree of variation is thus expected when generating dECM biomaterials from mammalian tissues. However, dECM biomaterials have frequently been shown to have batch‐to‐batch variation beyond that of native tissue.^[^ ^148^ ^]^ This is generally considered to be a by‐product of the decellularization processes, generating variation in the final DNA content and matrix compositional variations of dECM products referred to as batch‐to‐batch variability.^[^ ^149^ ^]^ Batch‐to‐batch variability remains a significant challenge in the generation of dECM biomaterials suitable for clinical use. Means to mitigate batch‐to‐batch variation include homogenizing tissue particulates to increase surface area and ensure consistent exposure of decellularization agent which in turn can reduce variation and exposure time required for successful decellularization.^[^ ^148^ ^]^ However, this approach still relies on end point measurement of the decellularization process. There is a strong and urgent need to identify, detect, and adjust process parameters during decellularization in order to correlate between decellularization parameters and final dECM biomaterial quality.

Regulatory agencies such as the FDA and EMA require extensive preclinical data, including toxicology studies, immunogenicity testing, and clinical trial results, before dECM materials can be approved for use. Critical to the development of dECM products is the standardization of methods to assess the regenerative potential and identify sterilization and storage modalities that retain maximal regenerative potential to enable clinical translation. Recently, the FDA has provided a guidance document (FDA‐2000‐D‐0129) regarding the use of xenotransplantation products in humans. This guidance sets forward the required testing and material characterization necessary for tissue‐derived materials. Rigorous standardized processes are needed to ensure efficient, homogenous, and reproducible processes in large‐scale manufacturing of dECM biomaterials.^[^ ^150^ ^]^

Manufacturers of dECM products often report processing protocols in order to standardize and register the processing and fabrication of clinically utilized dECM scaffolds.^[^ ^14^ ^]^ However, many challenges still exist in standardizing decellularization processes, especially across different tissue types. Given that each tissue has different physical characteristics, it is challenging to define standardized decellularization processes that are effective in cell removal while minimizing disruption to the biological entities present in the dECM. Although there are several laboratory studies that investigate standardized decellularization protocols using bioreactor systems,^[^ ^151^, ^152^ ^]^ to our knowledge, these have not been translated to the commercial setting. To meet the demands of clinical applications, advances in bioreactor systems and large‐scale decellularization processes are being developed to allow for mass production of dECM‐based scaffolds.^[^ ^153^, ^154^, ^155^ ^]^ These technologies are intended to improve consistency in manufacturing, reduce costs, and improve the accessibility of dECM biomaterials for widespread use. Ensuring the consistent quality and reproducibility of dECM materials across batches is essential for their clinical success. Variability in ECM properties, such as structure, composition, and bioactivity, can affect their performance in therapeutic applications. Automated systems to standardize decellularization for dECM biomaterial manufacturing have recently been developed and include the use of in‐line DNA removal tracing,^[^ ^149^ ^]^ automated reagent dosing,^[^ ^156^ ^]^ and a proof‐of‐concept study demonstrating a standardized multitissue decellularization protocol to derive dECM using an automated system.^[^ ^151^ ^]^ While these advancements are key to reducing batch‐to‐batch variation, they have largely been demonstrated in laboratory environments with small‐scale dECM batches (1 g tissue). The challenge now is to translate and upscale automated and in‐line process monitoring of large‐scale ECM manufacturing processes.

Regulatory hurdles of dECM products stem from the complex biological nature and the evolving understanding of therapeutic action of ECM‐derived biomaterials. One of the primary challenges is determining whether dECM biomaterials should be classified as biologics or medical devices. This distinction affects the regulatory pathway, approval process, and requirements for clinical use. Initially, dECM biomaterials were often regulated as medical devices with a classification of biologically inert materials.^[^ ^14^ ^]^ However, studies of dECM modulatory impacts on cell behaviors have led to a richer understanding of the biologic activity of dECM biomaterials.^[^ ^157^, ^158^ ^]^ As such, dECM biomaterials can be considered to have properties that meet the regulation considerations of both medical devices and functional biologics. Traditionally, the FDA regulated dECM products as medical devices and often followed a 510(k) regulatory pathway, where allowance is granted based on demonstrable similarity to a preapproved or predicate product. If there is no predicate device available for a specific application, dECM products must follow a premarket approval (PMA) pathway. Those dECM products that are considered to have biological functionality follow a biologics license application (BLA). This was the case recently for the first dECM hydrogel, VentriGel, utilized in myocardial infarction repair, which followed the BLA regulatory route due to the mechanism of action of the dECM biomaterial.^[^ ^33^ ^]^ To enable broader clinical translation and adoption of dECM biomaterials, researchers will need to generate robust mechanistic data to identify the therapeutic mode of action and engage regulatory bodies early to navigate the evolving distinctions between biologic and device classifications.

With the advent of artificial‐intelligence (AI)‐aided design in biomaterials, there is immense potential to improve process standardization and reproducibility by identifying potential applications of this technology. Providing AI with preclinical and clinical data could enable the establishment of patterns between tissue source and composition and resulting scaffold characteristics including bioactivity, toxicology, and immunogenicity. AI has the potential to elucidate important aspects of tissue characterization to predict variability in scaffold fabrication. This would enable the rapid downselection of tissue donors and a reduction in batch variability prior to clinical translation. Additionally, AI could be employed to determine the optimal polymeric additives for each fabrication type according to required mechanical properties of the final application. This would be a field‐specific use of AI and would require separate optimization for each fabrication type. For example, developing 3D printing models would require information on printing parameters and viscoelastic properties of the inks, while electrospinning models would require an understanding of solution viscosity and the chain entanglements necessary for fiber formation. Finally, AI could contribute to developing patient‐specific scaffolds based on the growth factors or compositional constituents best suited for a patient's needs. Including patient data in the design of a dECM scaffold could allow for the optimization of tissue source and scaffold structure to improve clinical outcomes. Overall, AI is an emerging platform that should be considered in the development of dECM biomaterials to improve the standardization of dECM fabrication and the regenerative potential of dECM scaffolds.

## 5 Summary

Scaffolds fabricated from dECM biomaterials hold significant promise for advancing tissue engineering and regenerative medicine. The regenerative potential of these tissue‐derived materials is unsurpassed with a wealth of tissue‐specific biochemical cues and structural molecules that improve remodeling and regeneration upon implantation. Manufacturing advances are poised to make dECM biomaterials more versatile, reproducible, and customizable. Advanced dECM manufacturing techniques including mold casting, electrodynamic processing, emulsion‐based fabrication, and additive manufacturing enable the development of highly complex and functional dECM scaffolds, paving the way for their broader use in clinical settings and advancing the field of regenerative medicine. Challenges remain in scaling these technologies for clinical adoption, especially in maintaining quality control, ensuring consistency across batches, and navigating an evolving regulatory environment. Addressing these challenges will unlock the full potential of dECM medical products in regenerative medicine, offering transformative solutions for tissue repair and regeneration.

## Conflict of Interest

The authors declare no conflict of interest.

## Supporting information



Supporting Information
