# Supplementary material for: Extracellular‐Matrix‐Based Materials from Decellularized Tissue: Opportunities, Challenges, and Future Directions in Regenerative Medicine
Source: Adv Healthc Mater. 2025 Nov 17;15(1):e02107. doi: 10.1002/adhm.202502107 (PMC12790312; doi:10.1002/adhm.202502107)
Supplement: Supplementary file 1 — Supporting Information [file ADHM-15-0-s001.docx]

**Extracellular Matrix-Based Materials from Decellularized Tissue: Opportunities, Challenges, and Future Directions in Regenerative Medicine**

*Madeline Laude, Vasiliki Kolliopoulos, Antonios G. Mikos, Lisa J. White,*

*Elizabeth Cosgriff-Hernandez^†^*

**Supplemental Information**

**Supplementary Table 1:** Summary of dECM hydrogel fabrication including tissue source, ECM form, content, post-processing steps, and additives.

| **Species** | **Tissue Source** | **ECM Form** | **Content** | **Post-Processing** | **Additive** |
| --- | --- | --- | --- | --- | --- |
| Porcine | Small Intestinal Submucosa | Digested | 10 mg/ml | Heat Gelation | None^1^ |
|  | Bone | Digested | 20 mg/ml | Heat Gelation | None^2^ |
|  | Muscle | Digested | 10 mg/ml | Heat Gelation | None^3^ |
|  | Pericardium | Digested | 10 mg/ml | Heat Gelation | None^4^ |
|  | Myocardium | Digested | 6 mg/ml | Heat Gelation | None^5^ |
|  | Brain | Digested | 20 mg/ml | Heat Gelation | None^6^ |
|  | Cardiac Muscle | Digested | 10 mg/ml | Heat Gelation | None^7^ |
|  | Liver | Digested | 0.5-0.75 mg/ml | Photo-Initiated Crosslinking | None^8^ |
|  |  | Digested | 10 mg/ml | Glutaraldehyde Crosslinking | None^9^ |
|  | Dermis | Digested | 2-8 mg/ml | Heat Gelation | None^10^ |
|  |  | Particulate | 25-100 mg/ml | Heat Gelation | None^11^ |
| Human | Umbilical Cord | Digested | 10 mg/ml | Heat Gelation | None^12^ |
|  | Lung | Digested | 20 mg/ml | Heat Gelation | None^13^ |
| Bovine | Ovaries | Digested | 10 mg/ml | Heat Gelation | None^14^ |
|  | Tendon | Digested | 30 mg/ml | Photo-Initiated Crosslinking | None^15^ |
|  | Cartilage | Digested | 30 mg/ml | Photo-Initiated Crosslinking | None^15^ |
| Rat | Liver | Digested | 40 mg/ml | Heat Gelation | None^16^ |

**Supplementary Table 2:** Summary of dECM casted foam fabrication including tissue source, ECM form, content, post-processing steps, additives, and solvents.

| **Species** | **Tissue Source** | **ECM Form** | **Content** | **Post-Processing** | **Additive** | **Solvent** |
| --- | --- | --- | --- | --- | --- | --- |
| Porcine | Heart | Digested | 20-50 mg/ml | Lyophilization | None | Acetic Acid^17^ |
|  | Adipose | Digested | 50 mg/ml | Lyophilization | None | Acetic Acid^18^ |
|  |  | Digested | 25, 50, 100 mg/ml | Lyophilization | None | Acetic Acid^19^ |
|  |  | Particulate | 0.5% w/v | Lyophilization | None | Acetic Acid^20^ |
| Human | Placenta | Digested | 0.05 g/ml | Lyophilization | Silk Fibroin | Water^21^ |
|  | Adipose | Digested | 25 mg/ml | Lyophilization | None | 𝛼-amylase^22^ |
|  |  | Particulate | 20 mg/ml | Lyophilization | None | Acetic Acid^23^ |
|  |  | Particulate | 66.7 wt% | Lyophilization | None | Water^24^ |

**Supplementary Table 3:**Summary of dECM electrospinning fabrication including tissue source, ECM form, content, post-processing steps, additives, and solvents.

| **Species** | **Tissue Source** | **ECM Form** | **Content** | **Post-Processing** | **Additive** | **Solvent** |
| --- | --- | --- | --- | --- | --- | --- |
| Porcine | Small Intestinal Submucosa | Digested | 0.5% w/v | None | PU | HFIP^25^ |
|  |  | Digested | 0.25-1 wt% | None | PLGA | HFIP^26^ |
|  |  | Particulate | 0.75-2.1 wt% | None | Silk Fibroin, PCL | Methylene Chloride/DMF^27^ |
|  |  | Particulate | 0.5-3 wt% | None | PCL | Methyl Chloride/DMF^28^ |
|  |  | Particulate | 5 wt% | None | PCL | HFIP^29^ |
|  | Cartilage | Digested | 10 wt% | None | PCL | HFIP^30^ |
|  |  | Particulate | 8% w/v | None | PCL | HFIP^31^ |
|  | Skeletal Muscle | Digested | 10% w/v | Photo-Initiated Crosslinking | None | HFIP^32^ |
|  | Meniscus | Particulate | 8% w/v | EDAC Crosslinking | PCL | HFIP^33^ |
|  | Lung | Particulate | 3.5% w/v | None | PLLA | HFIP^34^ |
|  | Pancreas | Particulate | 0.4% w/v | None | Silk Fibroin | HFIP^35^ |
| Human | Adipose | Digested | 6-14% w/v | Genipin Crosslinking | None | HFIP^36^ |
|  | Cartilage | Particulate | 6 wt% | Hydrolyzed with NaOH | PHB/PHBV | Chloroform/DMF^37^ |
|  | Adipose | Particulate | 6 wt% | Hydrolyzed with NaOH | PHB/PHBV | Chloroform/DMF^37^ |
| Rabbit | Skeletal Muscle | Particulate | 10% w/v | Glutaraldehyde Crosslinking | None | HFIP^38^ |
|  |  | Particulate | 10% w/v | Glutaraldehyde Crosslinking | None | HFIP^39^ |
| Bovine | Skeletal Muscle | Digested | 10% w/v | None | None | HFIP^40^ |
| Rat | Brain | Particulate | 1% w/v | Genipin Crosslinking | Gelatin | Acetic Acid/DI Water^41^ |

**Supplementary Table 4*:*** Summary of dECM electrospraying fabrication including tissue source, ECM form, content, post-processing steps, additives, and solvents.

| **Species** | **Tissue Source** | **ECM Form** | **Content** | **Post-Processing** | **Additive** | **Solvent** |
| --- | --- | --- | --- | --- | --- | --- |
| Human | Adipose | Digested | 25 mg/ml | Lyophilization | Collagen | 𝛼-amylase^22^ |
|  |  | Digested | 50 mg/ml | Lyophilization | None | Acetic Acid^18^ |
|  |  | Digested | 25-100 mg/ml | Lyophilization | None | Acetic Acid^19^ |
|  |  | Particulate | 20 mg/ml | Lyophilization | None | Acetic Acid^23^ |
| Porcine | Dermis | Digested | 25-100 mg/ml | Lyophilization | None | Acetic Acid^42^ |
|  | Heart | Digested | 25-100 mg/ml | Lyophilization | None | Acetic Acid^42^ |

**Supplementary Table 5:** Summary of dECM emulsion-based fabrication including tissue source, ECM form, content, post-processing steps, additives, solvents, and continuous phase.

| **Species** | **Tissue Source** | **ECM Form** | **Content** | **Post-Processing** | **Additive** | **Solvent** | **Continuous Phase** |
| --- | --- | --- | --- | --- | --- | --- | --- |
| Porcine | Nerve Tissue | Digested | 1% w/v | Heat Gelation | None | HCl, Pepsin, NaOH, PBS | Mineral Oil, Span 80^43^ |
|  | Skeletal Muscle | Digested | 3 wt% | Heat Gelation | Gelatin, PLCL | Acetic Acid, Pepsin, NaOH | PVA^44^ |
|  | Ventricles | Digested | 1% w/v | Heat Gelation | None | Digestion Buffer, NaOH | Olive Oil^45^ |
|  | Cartilage | Digested | 4% w/v | Heat Gelation | PLGA, Gelatin | HCl, Pepsin | PVA^46^ |
|  |  | Digested | 2 wt% | Photo-Initiated Crosslinking | HAMA | Acetic Acid, Pepsin | Paraffin Oil, Span 80^47^ |
| Human | Adipose | Digested | 2-4 mg/ml | Photo-Initiated Crosslinking | Alginate | Acetic Acid | CaCl_2_ solution^48^ |
|  |  | Digested | 1-4% w/v | Photo-Initiated Crosslinking | Alginate | Acetic Acid | CaCl_2_ solution^49^ |
| Rat | Bone | Digested | 1% w/v | Glutaraldehyde Crosslinking | None | Acetic Acid | Acetone^50^ |

**Supplementary Table 6:** Summary of dECM digital light processing (DLP) printing fabrication including tissue source, ECM form, content, post-processing steps, additives, and solvents.

| **Species** | **Tissue Source** | **ECM Form** | **Content** | **Post-Processing** | **Additive** | **Solvent** |
| --- | --- | --- | --- | --- | --- | --- |
| Porcine | Liver | Digested | 3% w/v | Photo-Initiated Crosslinking | GelMA | Acetic Acid, Pepsin, NaOH^51^ |
|  | Liver | Digested | 5% w/v | Photo-Initiated Crosslinking | GelMA | PBS^52^ |
|  | Cornea | Digested | 0.5-1% w/v | Photo-Initiated Crosslinking | GelMA | Acetic Acid, Pepsin, NaOH^53^ |
|  | Cartilage | Digested | 1-3% w/v | Photo-Initiated Crosslinking | GelMA, Exosomes | Acetic Acid, Pepsin^54^ |
|  | Tendon | Digested | 1% w/v | Photo-Initiated Crosslinking | PEGDA | Acetic Acid, Pepsin, NaOH^55^ |
|  | Heart | Digested | 5% w/ | Photo-Initiated Crosslinking | GelMA | PBS^52^ |
|  | Heart | Digested | 1% w/v | Photo-Initiated Crosslinking | Ru/SPS | Acetic Acid, Pepsin, NaOH, PBS^56^ |
| Bovine | Cornea | Digested | 1% w/v | Photo-Initiated Crosslinking | Ru/SPS | Acetic Acid, Pepsin, NaOH, PBS^56^ |
| Rat | Liver | Digested | 70 wt% | Photo-Initiated Crosslinking | PCL-MA | Formamide^57^ |

**Supplementary Table 7:** Summary of dECM extrusion 3D printing fabrication including tissue source, ECM form, content, post-processing steps, additives, and solvents.

| **Species** | **Tissue Source** | **ECM Form** | **Content** | **Post-Processing** | **Additive** | **Solvent** |
| --- | --- | --- | --- | --- | --- | --- |
| Porcine | Aorta | Digested | 3-10% w/v | CaCl_2_ | Gelatin, Alginate | DI Water^58^ |
|  | Cardiac Tissue | Digested | 33 mg/ml | Photo-Initiated Crosslinking | None | Pepsin, PBS, NaOH^59^ |
|  | Cardiac Tissue | Digested | 10% w/v | Photo-Initiated Crosslinking | PEG-DA, Nanoclay | DI Water^60^ |
|  | Skeletal Muscle | Digested | 1% w/v | Heat Gelation | PCL Framework | Pepsin, NaOH, PBS^61^ |
|  | Meniscus | Digested | 3-7% w/v | pH Gelation | Alginate | Growth Medium^62^ |
|  | Cartilage | Digested | 10-15% w/v | Photo-Initiated Crosslinking | Gelatin | Pepsin, Acetic Acid, PBS^63^ |
|  | Cartilage | Digested | 10% w/v | Heat Gelation | PCL | Acetic Acid, Pepsin, NaOH^64^ |
|  | Heart | Digested | 2.5% w/v | Heat Gelation | Collagen | Acetic Acid^65^ |
|  | Heart | Digested | 10% w/v | Heat Gelation | PCL | Pepsin, Acetic Acid, NaOH^64^ |
|  | Heart | Digested | 2% w/v | Heat Gelation | Vitamin B2 | Pepsin, Acetic Acid, PBS, NaOH^66^ |
|  | Liver | Particulate | 1-3% w/v | Heat Gelation | Gelatin, Fibrinogen | Hyaluronic Acid, 𝛼-MEM^67^ |
|  | Bone | Particulate | 0.62 mg/ml | Enzymatic Crosslinking | Gelatin, Fibrinogen, Fibronectin | PBS, Media^68^ |
|  | Cartilage | Particulate | 0.2 g/ml | Heat Gelation | Hyaluronic Acid | DPBS^69^ |
| Human | Adipose | Digested | 10% w/v | Heat Gelation | PCL Framework | DMEM, Pepsin, Acetic Acid, NaOH^70^ |
|  | Lung | Digested | 6-10% w/v | CaCl_2_ | Alginate | HCl, Pepsin, NaOH^71^ |
| Rat | Bone | Digested | 20% w/v | Photo-Initiated Crosslinking | Gelatin | PBS^50^ |

**References:**

(1) Giobbe, G. G.; Crowley, C.; Luni, C.; Campinoti, S.; Khedr, M.; Kretzschmar, K.; De Santis, M. M.; Zambaiti, E.; Michielin, F.; Meran, L.; et al. Extracellular matrix hydrogel derived from decellularized tissues enables endodermal organoid culture. *Nature Communications* **2019**, *10* (1), 5658. DOI: 10.1038/s41467-019-13605-4.

(2) Kang, H.-J.; Park, S.-S.; Tripathi, G.; Lee, B.-T. Injectable demineralized bone matrix particles and their hydrogel bone grafts loaded with β-tricalcium phosphate powder and granules: A comparative study. *Materials Today Bio* **2022**, *16* (100422). DOI: <https://doi.org/10.1016/j.mtbio.2022.100422>.

(3) Fu, Y.; Fan, X.; Tian, C.; Luo, J.; Zhang, Y.; Deng, L.; Qin, T.; Lv, W. Decellularization of porcine skeletal muscle extracellular matrix for the formulation of a matrix hydrogel: a preliminary study. *Journal of Cellular and Molecular Medicine* **2016**, *20* (4), 740-749. DOI: <https://doi.org/10.1111/jcmm.12776>.

(4) Seif-Naraghi, S. B.; Salvatore Ma Fau - Schup-Magoffin, P. J.; Schup-Magoffin Pj Fau - Hu, D. P.; Hu Dp Fau - Christman, K. L.; Christman, K. L. Design and characterization of an injectable pericardial matrix gel: a potentially autologous scaffold for cardiac tissue engineering. (1937-335X (Electronic)). From 2010 Jun.

(5) Singelyn, J. M.; Sundaramurthy P Fau - Johnson, T. D.; Johnson Td Fau - Schup-Magoffin, P. J.; Schup-Magoffin Pj Fau - Hu, D. P.; Hu Dp Fau - Faulk, D. M.; Faulk Dm Fau - Wang, J.; Wang J Fau - Mayle, K. M.; Mayle Km Fau - Bartels, K.; Bartels K Fau - Salvatore, M.; Salvatore M Fau - Kinsey, A. M.; et al. Catheter-deliverable hydrogel derived from decellularized ventricular extracellular matrix increases endogenous cardiomyocytes and preserves cardiac function post-myocardial infarction. (1558-3597 (Electronic)). From 2012 Feb 21.

(6) DeQuach, J. A.; Yuan Sh Fau - Goldstein, L. S. B.; Goldstein Ls Fau - Christman, K. L.; Christman, K. L. Decellularized porcine brain matrix for cell culture and tissue engineering scaffolds. (1937-335X (Electronic)). From 2011 Nov.

(7) Song, Y.; You, Y.; Xu, X.; Lu, J.; Huang, X.; Zhang, J.; Zhu, L.; Hu, J.; Wu, X.; Xu, X.; et al. Adipose-Derived Mesenchymal Stem Cell-Derived Exosomes Biopotentiated Extracellular Matrix Hydrogels Accelerate Diabetic Wound Healing and Skin Regeneration. *Advanced Science* **2023**, *10* (30), 2304023. DOI: <https://doi.org/10.1002/advs.202304023> (acccessed 2025/03/05).

(8) Ravichandran, A., Murekatete, B., Moedder, D. et al. Photocrosslinkable liver extracellular matrix hydrogels for the generation of 3D liver microenvironment models. *Sci Rep* **2021**, *11, 15566*. DOI: <https://doi.org/10.1038/s41598-021-94990-z>.

(9) Ijima, H.; Nakamura, S.; Bual, R. P.; Yoshida, K. Liver-specific extracellular matrix hydrogel promotes liver-specific functions of hepatocytes in vitro and survival of transplanted hepatocytes in vivo. *Journal of Bioscience and Bioengineering* **2019**, *128* (3), 365-372. DOI: <https://doi.org/10.1016/j.jbiosc.2019.02.014>.

(10) Wolf, M. T.; Daly Ka Fau - Brennan-Pierce, E. P.; Brennan-Pierce Ep Fau - Johnson, S. A.; Johnson Sa Fau - Carruthers, C. A.; Carruthers Ca Fau - D'Amore, A.; D'Amore A Fau - Nagarkar, S. P.; Nagarkar Sp Fau - Velankar, S. S.; Velankar Ss Fau - Badylak, S. F.; Badylak, S. F. A hydrogel derived from decellularized dermal extracellular matrix. (1878-5905 (Electronic)). From 2012 Oct.

(11) Hussey, G. S.; Nascari, D. G.; Saldin, L. T.; Kolich, B.; Lee, Y. C.; Crum, R. J.; El-Mossier, S. O.; D'Angelo, W.; Dziki, J. L.; Badylak, S. F. Ultrasonic cavitation to prepare ECM hydrogels. *Acta Biomaterialia* **2020**, *108*, 77-86. DOI: <https://doi.org/10.1016/j.actbio.2020.03.036>.

(12) Výborný, K.; Vallová, J.; Kočí, Z.; Kekulová, K.; Jiráková, K.; Jendelová, P.; Hodan, J.; Kubinová, Š. Genipin and EDC crosslinking of extracellular matrix hydrogel derived from human umbilical cord for neural tissue repair. *Scientific Reports* **2019**, *9* (1), 10674. DOI: 10.1038/s41598-019-47059-x.

(13) de Hilster, R. H. J.; Sharma, P. K.; Jonker, M. R.; White, E. S.; Gercama, E. A.; Roobeek, M.; Timens, W.; Harmsen, M. C.; Hylkema, M. N.; Burgess, J. K. Human lung extracellular matrix hydrogels resemble the stiffness and viscoelasticity of native lung tissue. *American Journal of Physiology-Lung Cellular and Molecular Physiology* **2020**, *318* (4), L698-L704. DOI: 10.1152/ajplung.00451.2019 (acccessed 2025/03/05).

(14) Chiti, M.-C.; Vanacker, J.; Ouni, E.; Tatic, N.; Viswanath, A.; des Rieux, A.; Dolmans, M.-M.; White, L. J.; Amorim, C. A. Ovarian extracellular matrix-based hydrogel for human ovarian follicle survival in vivo: A pilot work. *Journal of Biomedical Materials Research Part B: Applied Biomaterials* **2022**, *110* (5), 1012-1022. DOI: <https://doi.org/10.1002/jbm.b.34974> (acccessed 2025/03/05).

(15) Rothrauff, B. B.; Yang, G.; Tuan, R. S. Tissue-specific bioactivity of soluble tendon-derived and cartilage-derived extracellular matrices on adult mesenchymal stem cells. *Stem Cell Research and Therapy* **2017**, *8*. DOI: <https://doi.org/10.1186/s13287-017-0580-8>.

(16) Lee, J. S.; Shin, J.; Park, H.-M.; Kim, Y.-G.; Kim, B.-G.; Oh, J.-W.; Cho, S.-W. Liver Extracellular Matrix Providing Dual Functions of Two-Dimensional Substrate Coating and Three-Dimensional Injectable Hydrogel Platform for Liver Tissue Engineering. *Biomacromolecules* **2014**, *15* (1), 206-218. DOI: 10.1021/bm4015039.

(17) Russo, V.; Omidi, E.; Samani, A.; Hamilton, A.; Flynn, L. Porous, Ventricular Extracellular Matrix-Derived Foams as a Platform for Cardiac Cell Culture. *BioResearch Open Access* **2015**, *4*, 374-388. DOI: 10.1089/biores.2015.0030.

(18) Kornmuller, A.; Brown, C. F. C.; Yu, C.; Flynn, L. E. Fabrication of Extracellular Matrix-derived Foams and Microcarriers as Tissue-specific Cell Culture and Delivery Platforms. *JoVE* **2017**, (122), e55436. DOI: doi:10.3791/55436.

(19) Yu, C.; Bianco, J.; Brown, C.; Fuetterer, L.; Watkins, J. F.; Samani, A.; Flynn, L. E. Porous decellularized adipose tissue foams for soft tissue regeneration. *Biomaterials* **2013**, *34* (13), 3290-3302. DOI: <https://doi.org/10.1016/j.biomaterials.2013.01.056>.

(20) Luzuriaga, J.; García-Gallastegui, P.; García-Urkia, N.; Pineda, J. R.; Irastorza, I.; Fernandez-San-Argimiro, F. J.; Briz, N.; Olalde, B.; Unda, F.; Madarieta, I.; et al. Osteogenic differentiation of human dental pulp stem cells in decellularised adipose tissue solid foams. *Eur Cell Mater* **2022**, *43*, 112-129. DOI: 10.22203/eCM.v043a10 From NLM.

(21) Rameshbabu, A. P.; Bankoti, K.; Datta, S.; Subramani, E.; Apoorva, A.; Ghosh, P.; Jana, S.; Manchikanti, P.; Roy, S.; Chaudhury, K.; et al. Bioinspired 3D porous human placental derived extracellular matrix/silk fibroin sponges for accelerated bone regeneration. *Materials Science and Engineering: C* **2020**, *113*, 110990. DOI: <https://doi.org/10.1016/j.msec.2020.110990>.

(22) Morissette Martin, P.; Grant, A.; Hamilton, D. W.; Flynn, L. E. Matrix composition in 3-D collagenous bioscaffolds modulates the survival and angiogenic phenotype of human chronic wound dermal fibroblasts. *Acta Biomaterialia* **2019**, *83*, 199-210. DOI: <https://doi.org/10.1016/j.actbio.2018.10.042>.

(23) Morissette Martin, P.; Walker, J. T.; Kim, K. J.; Brooks, C. R.; Serack, F. E.; Kornmuller, A.; Juignet, L.; Hamilton, A. M.; Dunmore-Buyze, P. J.; Drangova, M.; et al. Modular cell-assembled adipose matrix-derived bead foams as a mesenchymal stromal cell delivery platform for soft tissue regeneration. *Biomaterials* **2021**, *275*, 120978. DOI: <https://doi.org/10.1016/j.biomaterials.2021.120978>.

(24) Lee, Y. J.; Baek, S. E.; Lee, S.; Jeong, Y. J.; Kim, K. J.; Jun, Y. J.; Rhie, J. W. Wound-healing effect of adipose stem cell-derived extracellular matrix sheet on full-thickness skin defect rat model: Histological and immunohistochemical study. *International Wound Journal* **2019**, *16* (1), 286-296. DOI: <https://doi.org/10.1111/iwj.13030> (acccessed 2025/03/05).

(25) Kim, K.; Kim, J.; Kwak, S.-W.; Lee, J. Y.; Kim, H.-Y. Electrospun Polyurethane/Small Intestinal Submucosa Blended Nanofibrous Mats for Potential Wound Healing. *Fibers and Polymers* **2022**, *23* (9), 2557-2564. DOI: 10.1007/s12221-022-4918-1.

(26) Kim, K.; Lee, J. Y.; Kim, H.; Shin, J.; Shin, Y.; Yoo, Y. T.; Kim, H.-Y. Fabrication of electropsun PLGA and small intestine submucosa-blended nanofibrous membranes and their biocompatibility for wound healing. *Fibers and Polymers* **2017**, *18* (2), 231-239. DOI: 10.1007/s12221-017-6936-y.

(27) Hong, S.; Kim, G. H. Electrospun Polycaprolactone/Silk Fibroin/Small Intestine Submucosa Composites for Biomedical Applications. *Macromolecular Materials and Engineering* **2010**, *295* (6), 529-534. DOI: <https://doi.org/10.1002/mame.201000051> (acccessed 2025/07/23).

(28) Hong, S.; Kim, G. Electrospun micro/nanofibrous conduits composed of poly(epsilon-caprolactone) and small intestine submucosa powder for nerve tissue regeneration. (1552-4981 (Electronic)). From 2010 Aug.

(29) Jones, S.; VandenHeuvel, S.; Luengo Martinez, A.; Birur, R.; Burgeson, E.; Gilbert, I.; Baker, A.; Wolf, M.; Raghavan, S. A.; Rogers, S.; et al. Suspension electrospinning of decellularized extracellular matrix: A new method to preserve bioactivity. *Bioactive Materials* **2024**, *41*, 640-656. DOI: <https://doi.org/10.1016/j.bioactmat.2024.08.012>.

(30) Feng, B.; Ji, T.; Wang, X.; Fu, W.; Ye, L.; Zhang, H.; Li, F. Engineering cartilage tissue based on cartilage-derived extracellular matrix cECM/PCL hybrid nanofibrous scaffold. *Materials & Design* **2020**, *193*, 108773. DOI: <https://doi.org/10.1016/j.matdes.2020.108773>.

(31) Garrigues, N. W.; Little, D.; Sanchez-Adams, J.; Ruch, D. S.; Guilak, F. Electrospun cartilage-derived matrix scaffolds for cartilage tissue engineering. *Journal of Biomedical Materials Research Part A* **2014**, *102* (11), 3998-4008. DOI: <https://doi.org/10.1002/jbm.a.35068> (acccessed 2025/03/05).

(32) Lee, H.; Kim, W.; Lee, J.; Yoo, J. J.; Kim, G. H.; Lee, S. J. Effect of Hierarchical Scaffold Consisting of Aligned dECM Nanofibers and Poly(lactide-co-glycolide) Struts on the Orientation and Maturation of Human Muscle Progenitor Cells. *ACS Applied Materials & Interfaces* **2019**, *11* (43), 39449-39458. DOI: 10.1021/acsami.9b12639.

(33) Gao, G.; Lee, J. H.; Jang, J.; Lee, D. H.; Kong, J.-S.; Kim, B. S.; Choi, Y.-J.; Jang, W. B.; Hong, Y. J.; Kwon, S.-M.; et al. Tissue Engineered Bio-Blood-Vessels Constructed Using a Tissue-Specific Bioink and 3D Coaxial Cell Printing Technique: A Novel Therapy for Ischemic Disease. *Advanced Functional Materials* **2017**, *27* (33), 1700798. DOI: <https://doi.org/10.1002/adfm.201700798> (acccessed 2025/03/05).

(34) Young, B. M.; Shankar, K.; Allen, B. P.; Pouliot, R. A.; Schneck, M. B.; Mikhaiel, N. S.; Heise, R. L. Electrospun Decellularized Lung Matrix Scaffold for Airway Smooth Muscle Culture. *ACS Biomaterials Science & Engineering* **2017**, *3* (12), 3480-3492. DOI: 10.1021/acsbiomaterials.7b00384.

(35) Zhu, Y.; Wang, D.; Yao, X.; Wang, M.; Zhao, Y.; Lu, Y.; Wang, Z.; Guo, Y. Biomimetic hybrid scaffold of electrospun silk fibroin and pancreatic decellularized extracellular matrix for islet survival. *Journal of Biomaterials Science, Polymer Edition* **2021**, *32* (2), 151-165. DOI: 10.1080/09205063.2020.1818018.

(36) Francis, M. P.; Sachs, P. C.; Madurantakam, P. A.; Sell, S. A.; Elmore, L. W.; Bowlin, G. L.; Holt, S. E. Electrospinning adipose tissue-derived extracellular matrix for adipose stem cell culture. *Journal of Biomedical Materials Research Part A* **2012**, *100A* (7), 1716-1724. DOI: <https://doi.org/10.1002/jbm.a.34126> (acccessed 2025/03/05).

(37) Masaeli, E.; Karamali, F.; Loghmani, S.; Eslaminejad, M. B.; Nasr-Esfahani, M. H. Bio-engineered electrospun nanofibrous membranes using cartilage extracellular matrix particles. *Journal of Materials Chemistry B* **2017**, *5* (4), 765-776, 10.1039/C6TB02015A. DOI: 10.1039/C6TB02015A.

(38) Hogan, K. J.; Smoak, M. M.; Koons, G. L.; Perez, M. R.; Bedell, M. L.; Jiang, E. Y.; Young, S.; Mikos, A. G. Bioinspired electrospun decellularized extracellular matrix scaffolds promote muscle regeneration in a rat skeletal muscle defect model. *Journal of Biomedical Materials Research Part A* **2022**, *110* (5), 1090-1100. DOI: <https://doi.org/10.1002/jbm.a.37355> (acccessed 2025/03/05).

(39) Smoak, M. M.; Han, A.; Watson, E.; Kishan, A.; Grande-Allen, K. J.; Cosgriff-Hernandez, E.; Mikos, A. G. Fabrication and Characterization of Electrospun Decellularized Muscle-Derived Scaffolds. *Tissue Engineering Part C: Methods* **2019**, *25* (5), 276-287. DOI: 10.1089/ten.tec.2018.0339 (acccessed 2025/03/05).

(40) Patel, K. H.; Dunn, A. J.; Talovic, M.; Haas, G. J.; Marcinczyk, M.; Elmashhady, H.; Kalaf, E. G.; Sell, S. A.; Garg, K. Aligned nanofibers of decellularized muscle ECM support myogenic activity in primary satellite cells in vitro. *Biomedical Materials* **2019**, *14* (3), 035010. DOI: 10.1088/1748-605X/ab0b06.

(41) Baiguera, S.; Del Gaudio, C.; Lucatelli, E.; Kuevda, E.; Boieri, M.; Mazzanti, B.; Bianco, A.; Macchiarini, P. Electrospun gelatin scaffolds incorporating rat decellularized brain extracellular matrix for neural tissue engineering. *Biomaterials* **2014**, *35* (4), 1205-1214. DOI: <https://doi.org/10.1016/j.biomaterials.2013.10.060>.

(42) Kornmuller, A.; Flynn, L. E. Development and characterization of matrix-derived microcarriers from decellularized tissues using electrospraying techniques. *Journal of Biomedical Materials Research Part A* **2022**, *110* (3), 559-575. DOI: <https://doi.org/10.1002/jbm.a.37306> (acccessed 2025/06/23).

(43) Lin, Z.; Rao, Z.; Chen, J.; Chu, H.; Zhou, J.; Yang, L.; Quan, D.; Bai, Y. Bioactive Decellularized Extracellular Matrix Hydrogel Microspheres Fabricated Using a Temperature-Controlling Microfluidic System. *ACS Biomaterials Science & Engineering* **2022**, *8* (4), 1644-1655. DOI: 10.1021/acsbiomaterials.1c01474.

(44) Li, Y., Liu, S., Zhang, J. et al. Elastic porous microspheres/extracellular matrix hydrogel injectable composites releasing dual bio-factors enable tissue regeneration. *Nat Commun* **2024**, *15* (1377). DOI: <https://doi.org/10.1038/s41467-024-45764-4>.

(45) Wang, X.; Ansari, A.; Pierre, V.; Young, K.; Kothapalli, C. R.; von Recum, H. A.; Senyo, S. E. Injectable Extracellular Matrix Microparticles Promote Heart Regeneration in Mice with Post-ischemic Heart Injury. *Advanced Healthcare Materials* **2022**, *11* (8), 2102265. DOI: <https://doi.org/10.1002/adhm.202102265> (acccessed 2025/03/05).

(46) Chen, Y.; Chen, L.-F.; Wang, Y.; Duan, Y.-Y.; Luo, S.-C.; Zhang, J.-T.; Kankala, R. K.; Wang, S.-B.; Chen, A.-Z. Modeling dECM-based inflammatory cartilage microtissues in vitro for drug screening. *Composites Part B: Engineering* **2023**, *250*, 110437. DOI: <https://doi.org/10.1016/j.compositesb.2022.110437>.

(47) Deng, S.; Cao, H.; Lu, Y.; Shi, W.; Chen, M.; Cui, X.; Liang, J.; Fan, Y.; Wang, Q.; Zhang, X. Injectable dECM-enhanced hyaluronic microgels with spatiotemporal release of cartilage-specific molecules to improve osteoarthritic chondrocyte’s function. *Collagen and Leather* **2024**, *6* (1), 14. DOI: 10.1186/s42825-024-00158-6.

(48) Allison E.B. Turner, C. Y., Juares Bianco, John F. Watkins, Lauren E. Flynn. The performance of decellularized adipose tissue microcarriers as an inductive substrate for human adipose-derived stem cells. *Biomaterials* **2012**, *33* (18), 4490-4499. DOI: <https://doi.org/10.1016/j.biomaterials.2012.03.026>.

(49) Turner, A. E. B.; Flynn, L. E. Design and Characterization of Tissue-Specific Extracellular Matrix-Derived Microcarriers. *Tissue Engineering Part C: Methods* **2011**, *18* (3), 186-197. DOI: 10.1089/ten.tec.2011.0246 (acccessed 2025/07/02).

(50) Hogan, K. J.; Öztatlı, H.; Perez, M. R.; Si, S.; Umurhan, R.; Jui, E.; Wang, Z.; Jiang, E. Y.; Han, S. R.; Diba, M.; et al. Development of photoreactive demineralized bone matrix 3D printing colloidal inks for bone tissue engineering. (2056-3418 (Print)). From 2023.

(51) Mao, Q.; Wang, Y.; Li, Y.; Juengpanich, S.; Li, W.; Chen, M.; Yin, J.; Fu, J.; Cai, X. Fabrication of liver microtissue with liver decellularized extracellular matrix (dECM) bioink by digital light processing (DLP) bioprinting. *Materials Science and Engineering: C* **2020**, *109*, 110625. DOI: <https://doi.org/10.1016/j.msec.2020.110625>.

(52) Yu, C.; Ma, X.; Zhu, W.; Wang, P.; Miller, K. L.; Stupin, J.; Koroleva-Maharajh, A.; Hairabedian, A.; Chen, S. Scanningless and continuous 3D bioprinting of human tissues with decellularized extracellular matrix. *Biomaterials* **2019**, *194*, 1-13. DOI: <https://doi.org/10.1016/j.biomaterials.2018.12.009>.

(53) Zhang, M.; Yang, F.; Han, D.; Zhang, S. Y.; Dong, Y.; Li, X.; Ling, L.; Deng, Z.; Cao, X.; Tian, J.; et al. 3D bioprinting of corneal decellularized extracellular matrix: GelMA composite hydrogel for corneal stroma engineering. (2424-8002 (Electronic)). From 2023.

(54) Chen, P.; Zheng, L.; Wang, Y.; Tao, M.; Xie, Z.; Xia, C.; Gu, C.; Chen, J.; Qiu, P.; Mei, S.; et al. Desktop-stereolithography 3D printing of a radially oriented extracellular matrix/mesenchymal stem cell exosome bioink for osteochondral defect regeneration. (1838-7640 (Electronic)). From 2019.

(55) Luo, Y.; Pan, H.; Jiang, J.; Zhao, C.; Zhang, J.; Chen, P.; Lin, X.; Fan, S. Desktop-Stereolithography 3D Printing of a Polyporous Extracellular Matrix Bioink for Bone Defect Regeneration. *Frontiers in Bioengineering and Biotechnology* **2020**, *8*. DOI: <https://doi.org/10.3389/fbioe.2020.589094>.

(56) Kim, H.; Kang, B.; Cui, X.; Lee, S.-H.; Lee, K.; Cho, D.-W.; Hwang, W.; Woodfield, T. B. F.; Lim, K. S.; Jang, J. Light-Activated Decellularized Extracellular Matrix-Based Bioinks for Volumetric Tissue Analogs at the Centimeter Scale. *Advanced Functional Materials* **2021**, *31* (32), 2011252. DOI: <https://doi.org/10.1002/adfm.202011252> (acccessed 2025/07/09).

(57) Elomaa, L.; Keshi, E.; Sauer, I. M.; Weinhart, M. Development of GelMA/PCL and dECM/PCL resins for 3D printing of acellular in vitro tissue scaffolds by stereolithography. *Materials Science and Engineering: C* **2020**, *112*. DOI: <https://doi.org/10.1016/j.msec.2020.110958>.

(58) Potere, F.; Belgio, B.; Croci, G. A.; Tabano, S.; Petrini, P.; Dubini, G.; Boschetti, F.; Mantero, S. 3D bioprinting of multi-layered segments of a vessel-like structure with ECM and novel derived bioink. *Frontiers in Bioengineering and Biotechnology* **2022**, *10*, Original Research.

(59) Jang, J.; Kim, T. G.; Kim, B. S.; Kim, S.-W.; Kwon, S.-M.; Cho, D.-W. Tailoring mechanical properties of decellularized extracellular matrix bioink by vitamin B2-induced photo-crosslinking. *Acta Biomaterialia* **2016**, *33*, 88-95. DOI: <https://doi.org/10.1016/j.actbio.2016.01.013>.

(60) Shin, Y. J.; Shafranek, R. T.; Tsui, J. H.; Walcott, J.; Nelson, A.; Kim, D.-H. 3D bioprinting of mechanically tuned bioinks derived from cardiac decellularized extracellular matrix. *Acta Biomaterialia* **2021**, *119*, 75-88. DOI: <https://doi.org/10.1016/j.actbio.2020.11.006>.

(61) Choi, Y.-J.; Kim, T. G.; Jeong, J.; Yi, H.-G.; Park, J. W.; Hwang, W.; Cho, D.-W. 3D Cell Printing of Functional Skeletal Muscle Constructs Using Skeletal Muscle-Derived Bioink. *Advanced Healthcare Materials* **2016**, *5* (20), 2636-2645. DOI: <https://doi.org/10.1002/adhm.201600483> (acccessed 2025/07/02).

(62) Porzucek, F.; Mankowska, M.; Semba, J. A.; Cywoniuk, P.; Augustyniak, A.; Mleczko, A. M.; Teixeira, A. M.; Martins, P.; Mieloch, A. A.; Rybka, J. D. Development of a porcine decellularized extracellular matrix (DECM) bioink for 3D bioprinting of meniscus tissue engineering: formulation, characterisation and biological evaluation. *Virtual and Physical Prototyping* **2024**, *19* (1), e2359620. DOI: 10.1080/17452759.2024.2359620.

(63) Hogan, K. J.; Perez, M. R.; Öztatlı, H.; Si, S.; Wang, Z.; Jiang, E. Y.; Diba, M.; Garipcan, B.; Mikos, A. G. Development of 3D-printing composite inks based on photoreactive cartilage extracellular matrix and gelatin nanoparticles. *Bioprinting* **2023**, *36*. DOI: <https://doi.org/10.1016/j.bprint.2023.e00317>.

(64) Pati, F. J., J. Ha, DH. et al. Printing three-dimensional tissue analogues with decellularized extracellular matrix bioink. *Nature Communications* **2014**, *5* (3935). DOI: <https://doi.org/10.1038/ncomms4935>.

(65) Das, S.; Kim, S.-W.; Choi, Y.-J.; Lee, S.; Lee, S.-H.; Kong, J.-S.; Park, H.-J.; Cho, D.-W.; Jang, J. Decellularized extracellular matrix bioinks and the external stimuli to enhance cardiac tissue development in vitro. *Acta Biomaterialia* **2019**, *95*, 188-200. DOI: <https://doi.org/10.1016/j.actbio.2019.04.026>.

(66) Jang, J.; Park, H.-J.; Kim, S.-W.; Kim, H.; Park, J. Y.; Na, S. J.; Kim, H. J.; Park, M. N.; Choi, S. H.; Park, S. H.; et al. 3D printed complex tissue construct using stem cell-laden decellularized extracellular matrix bioinks for cardiac repair. *Biomaterials* **2017**, *112*, 264-274. DOI: <https://doi.org/10.1016/j.biomaterials.2016.10.026>.

(67) Kim, M. K.; Jeong, W.; Lee, S. M.; Kim, J. B.; Jin, S.; Kang, H.-W. Decellularized extracellular matrix-based bio-ink with enhanced 3D printability and mechanical properties. *Biofabrication* **2020**, *12* (2), 025003. DOI: 10.1088/1758-5090/ab5d80.

(68) Moss, S. M.; Ortiz-Hernandez, M.; Levin, D.; Richburg, C. A.; Gerton, T.; Cook, M.; Houlton, J. J.; Rizvi, Z. H.; Goodwin, P. C.; Golway, M.; et al. A Biofabrication Strategy for a Custom-Shaped, Non-Synthetic Bone Graft Precursor with a Prevascularized Tissue Shell. *Frontiers in Bioengineering and Biotechnology* **2022**, *10*. DOI: <https://doi.org/10.3389/fbioe.2022.838415>.

(69) Jeanne E Barthold, K. P. M., Jaylene Martinez, Charlotte Bellerjeau, Yifu Ding, Stephanie J Bryant, Gregory L Whiting and Corey P Neu. Particulate ECM biomaterial ink is 3D printed and naturally crosslinked to form structurally-layered and lubricated cartilage tissue mimics. *Biofabrication* **2022**, *14*. DOI: 10.1088/1758-5090/ac584c.

(70) Pati, F.; Ha, D.-H.; Jang, J.; Han, H. H.; Rhie, J.-W.; Cho, D.-W. Biomimetic 3D tissue printing for soft tissue regeneration. *Biomaterials* **2015**, *62*, 164-175. DOI: <https://doi.org/10.1016/j.biomaterials.2015.05.043>.

(71) De Santis, M. M.; Alsafadi, H. N.; Tas, S.; Bölükbas, D. A.; Prithiviraj, S.; Da Silva, I. A. N.; Mittendorfer, M.; Ota, C.; Stegmayr, J.; Daoud, F.; et al. Extracellular-Matrix-Reinforced Bioinks for 3D Bioprinting Human Tissue. *Advanced Materials* **2021**, *33* (3), 2005476. DOI: <https://doi.org/10.1002/adma.202005476> (acccessed 2025/03/05).
